# Yiqi Wenyang Formula ameliorates diabetic kidney disease via inhibiting inflammation and regulating the gut microbiota-bile acid axis in mice by FXR signaling pathway

**DOI:** 10.1186/s13020-025-01238-3

**Published:** 2025-10-30

**Authors:** Tongyi Yuan, Xiang Gao, Xinxin Wang, Xiaolei Tang, Zuguo Liang, Yifei Yin, Songyan Liu, Jianze Ou, Wenjie Su, Zepeng Zhang, Xiangyan Li, Qingxia Huang, Daqing Zhao, He Zhang

**Affiliations:** 1 Research Center of Traditional Chinese Medicine, The Affiliated Hospital to Changchun University of Chinese Medicine, Changchun, 130021 China; 2 College of Pharmacy, Changchun University of Chinese Medicine, Changchun, 130117 China; 3 Northeast Asia Research Institute of Traditional Chinese Medicine, Changchun University of Chinese Medicine, Changchun, 130117 China

**Keywords:** Yiqi Wenyang Formula, diabetic kidney disease, gut microbiota, Bile acid, FXR, Molecular docking

## Abstract

**Background:**

Diabetic kidney disease (DKD) is a microangiopathic complication of diabetes. Yiqi Wenyang Formula (YQWYF) has been used to treat DKD in the clinic for many years. However, the underlying regulatory mechanisms of YQWYF on the gut microbiota and bile acids of DKD mice remain unclear.

**Purpose of the research:**

This study aimed to investigate the mechanism of YQWYF on DKD mice using an integrative approach of network pharmacology, fecal 16S ribosomal RNA (rRNA) gene sequencing, and untargeted metabolomics.

**Methods:**

The chemical composition of YQWYF was determined via ultrahigh-performance liquid chromatography/quadrupole time-of-flight mass spectrometry (UPLC-Q-TOF/MS). Common targets were identified between YQWYF and DKD, and a potential protein–protein interaction (PPI) network was constructed using network pharmacology. DKD mice were induced with streptozotocin (STZ) for 18 weeks. Twenty-four-hour urine was collected on the 9th and 18th weeks, and serum was collected on the 18th week to detect the biochemical measurements of urine and serum. Oxidative stress biomarkers and inflammatory cytokines in the kidney were detected via ELISA kits. The microstructure of the renal tissue was assessed by hematoxylin–eosin staining, periodic-acid Schiff staining, and Masson staining. Fresh fecal sample of mice were collected on the 18th week to detect the gut metabolites and microbiota using untargeted metabolomics and 16S rRNA sequencing. We analyzed the gut microbiota-bile acid (BA) axis for mechanism exploration.

**Results:**

A total of 41 compounds were recognized in YQWYF. TNF and IL6 were the important core targets between YQWYF and DKD. The results of molecular docking revealed that astragaloside I, 16-meprednisone acetate, and astragaloside IV from YQWYF had strong affinities for TNF-α and IL-6. Animal experiments showed that YQWYF reduced glycemia and improved lipid metabolism abnormalities in DKD mice. Moreover, it had excellent anti-oxidant and anti-inflammatory effects to ameliorate renal injury in DKD mice. YQWYF improved the richness and evenness of the gut microbiota and increased bile acid levels in the feces of DKD mice. Importantly, 4 genus bacteria (*christensenellaceae_R-7_group*, *oscillibacter*, *UCG-005*, and *[Eubacerium]_ xylanophilum_group*) were closely related with 3 BAs (CA, GCA and DHCA). Meanwhile, YQWYF improved the protein expression of Farnesoid X receptor (FXR), CYP7A1 and CYP8B in the liver.

**Conclusion:**

These findings reveal that YQWYF ameliorates renal injury in DKD mice by inhibiting the inflammatory, increasing the FXR signaling, and regulating the gut microbiota disorder and bile acid dysregulation.

**Graphical abstract:**

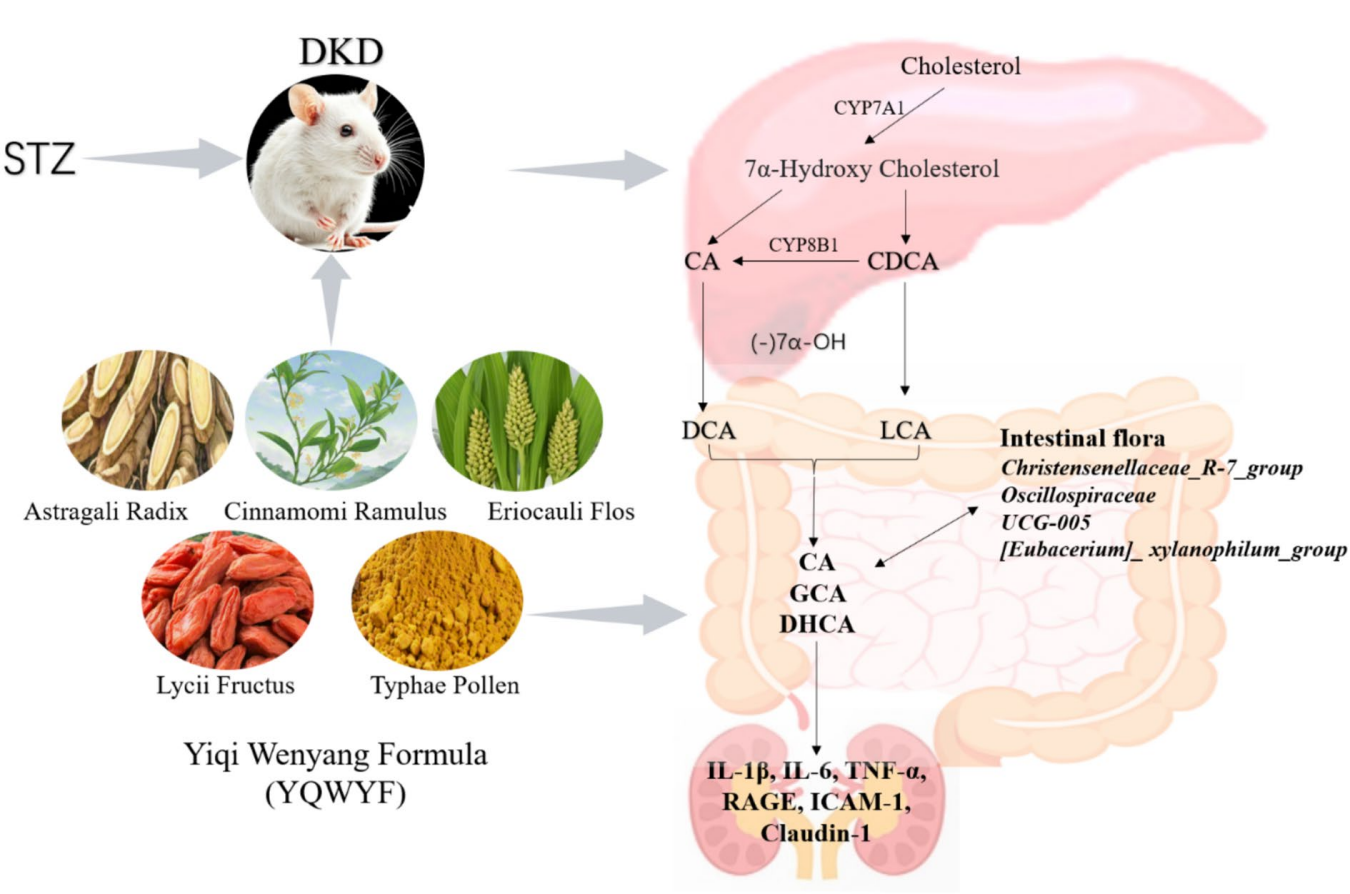

**Supplementary Information:**

The online version contains supplementary material available at 10.1186/s13020-025-01238-3.

## Introduction

Diabetes mellitus (DM) and its complications are becoming increasingly serious and threaten human health because of persistent hyperglycemia. Approximately 30% of patients with type 1 diabetes (T1D) and 40% of patients with type 2 diabetes (T2D) eventually develop diabetic kidney disease (DKD) [1]. DKD is a microvascular complication of DM that occurs in a high-sugar environment. The pathogenic mechanisms of DKD are mainly attributed to changes in hemodynamics and vascular structure [2]. Hyperglycemia, inflammatory cytokines, and oxidative stress cause microvascular endothelial impairment, vascular basement membrane thickening, platelet and red blood cell aggregation, and microthrombosis, which give rise to renal ischemia, nephron loss, and DKD progression, ultimately resulting in renal failure [3]. Because of ever increasing morbidity, mortality and high costs, thus, early prevention and intervention of DKD occurrence and development are important.

According to traditional Chinese medicine, DKD is accompanied by qi deficiency and blood stasis [4, 5]. The Yiqi Wenyang Formula (YQWYF) possesses ‘Qi boosting and stasis clearance’, which consisted of *Astragalus membranaceus* (Fisch.) Bunge (Astragali Radix, AR), *Lycium barbarum* L. (Lycii Fructus, LF), *Eriocaulon buergerianum* Koern. (Eriocauli Flos, EF), *Cinnamomum cassia* Presl (Cinnamomi Ramulus, CR), and *Typha angustifolia* L. (Typhae Pollen, TP). Recent clinical research has demonstrated that YQWYF effectively reduced blood sugar levels and improved microangiopathy in DM patients, including DKD, diabetic retinopathy (DR), and diabetic neuropathy [6, 7]. Modern pharmacological studies have shown that YQWYF improves renal injury and delays the progression of diabetic retinopathy in DKD and DR mice by exerting anti-oxidative, anti-inflammatory, and hypoglycemic effects [8, 9]. DM is a typical metabolic disease that causes abnormal metabolism of sugars, fat, proteins, and other substances, while changing the micro-ecological environment of the intestinal flora and causing pathological damage to body tissues or organs [10]. YQWYF improves microangiopathy, vascular endothelial dysfunction, and inflammation, thereby delaying diabetes development [8, 11]. However, the underlying mechanisms of YQWYF on regulating the gut microbiota and bile acid in DKD mice remain unclear. Calcium dobesilate (CaD) is used to treat microvascular complications of diabetes, which ameliorates hemodynamics, and restrains inflammatory molecules and renal fibrosis by reducing oxidative stress and the activation of MAPK and NF-κB [12, 13]. A recent clinical study also demonstrated that CaD reduced proteinuria in DKD [14]. Therefore, CaD was employed as a positive control in the experiment.

In this study, we investigated the mechanism of YQWYF in the treatment of DKD. First, the chemical profiles of YQWYF were determined via ultrahigh-performance liquid chromatography/quadrupole time-of-flight mass spectrometry (UPLC-Q-TOF/MS). Second, the therapeutic targets of YQWYF in DKD were predicted via network pharmacology. Third, we established DKD mice by intraperitoneal injection of streptozotocin (STZ). Finally, conjoint analysis of the gut metabolites and microbiota revealed the regulatory mechanism of YQWYF in DKD mice via untargeted metabolomics and 16S rRNA sequencing. Notably, we found that YQWYF improved renal injury in DKD mice by attenuating inflammation and regulating the gut microbiota-bile acid axis. Our findings provide new perspectives for preventing and delaying the occurrence and development of DKD.

## Materials and methods

### Preparation of YQWYF

Yiqi Wenyang Formula (YQWYF) was supplied from the Affiliated Hospital to Changchun University of Chinese Medicine (Changchun, Jilin). YQWYF includes *Astragalus membranaceus* (Fisch.) Bunge (Astragali Radix, AR), *Lycium barbarum* L. (Lycii Fructus, LF), *Eriocaulon buergerianum* Koern. (Eriocauli Flos, EF), *Cinnamomum cassia* Presl (Cinnamomi Ramulus, CR), and *Typha angustifolia* L. (Typhae pollen, TP). The ratio value of AR, LF, EF, CR and TP is 6:3:3:2:2. YQWYF was prepared using boiling water. Briefly, dried powders from 10 packages of YQWFY were soaked in 10 volumes of water for 4 h and then boiled three times for 2.5 h each time. All the supernatants were combined and concentrated by heating, and the concentrated solution was centrifuged (5,000 × *g* for 20 min). Finally, the supernatant of extract was freeze-dried to obtain YQWYF.

### Chemical composition detection

The chemical composition of YQWYF was determined via UPLC-Q-TOF/MS. Chromatographic separation was performed on an ACQUITY UPLC BEH C18 column (2.1 mm × 100 mm, 1.7 µm, Waters, USA) at 30 °C. The mobile phase included acetonitrile (A) and 0.1% formic acid (B). The gradient elution conditions were as follows: 0–4 min, 1% A; 4–7 min, 1–13% A; 7–12 min, 13–21% A; 12–14 min, 21–35% A; 14–18 min, 35–37% A; 18–21 min, 37–65% A; 21–24 min, 65–85% A; 24–26 min, 85% A; 26–28 min, 85–95% A; a flow rate was 0.3 mL/min, and the injection volume was 3 µL. High-accuracy MS data were recorded on a Waters Vion™ ion mobility quadrupole time-of-flight (IM-QTOF) MS (Waters, Manchester, UK) coupled with a UPLC I-Class system via a Zspray™ ESI source using the data-independent MS^E^ in the positive and negative ions. The ESI source parameters were as follows: capillary voltage, 2.5 kV; cone voltage, 60 V; collision energy, 40–80 eV; full-scan data, 100–1500 m*/z*; source temperature, 120 °C; desolvation temperature, 500 °C; cone gas flow rate, 50 L/h; and desolvation gas flow rate, 800 L/h [15].

UNIFI software (version 1.9.3.0, Waters) was used for data correction, peak picking, and peak annotation. The UNIFI parameters were as follows: the high-energy intensity threshold was 100.0 counts, the low-energy intensity threshold was 50.0 counts, and the target match tolerance was 10.0 ppm. The negative adducts include + HCOO, –H, + Cl, and + e. Lock mass combines width with three scans; the mass window was 0.5 m/z, the reference mass was 554.2620, and the reference charge was − 1 [15]. Meanwhile, 41 compounds were verified by the reference standards.

### Prediction of disease-related targets using Network pharmacology analysis of YQWYF for treating DKD

The SMLLE names of 33 components among 41 chemical components were searched in the PubChem database (https://pubchem.ncbi.nlm.nih.gov/) and then imported into the SwissTargetPrediction database (http://swisstargetprediction.ch) to obtain DKD’s corresponding targets of DKD. The GeneCards database (https://www.genecards.org/) was used to search for disease-related targets via the keyword “DKD” [16].

### Protein–protein interaction (PPI) network analysis

The therapeutic targets between YQWYF and DKD were constructed for visualization via a Venn diagram (https://bioinformatics.psb.ugent.be/webtools/Venn/). Therapeutic targets were analyzed via the STRING 11.5 database (https://string-db.org/) to generate a protein–protein interaction (PPI) network. The PPI network was visualized via Cytoscape 3.7.2 software [17].

### Gene Ontology (GO) and Kyoto Encyclopedia of Genes and Genomes (KEGG) enrichment analysis of the overlapping targets

Overlapping targets between YQWYF and DKD were imported into the Database for Annotation, Visualization, and Integrated Discovery (DAVID) (https://david.ncifcrf.gov/), and then, GO and KEGG enrichment analysis was performed. The bioinformatics visualization was created using the Metascape database (https://metascape.org/) with an adjusted *p* value ≤ 0.05 [17].

### Molecular docking verification

2D or 3D structures of the 33 compounds from YQWYF were downloaded from the PubChem database. The 3D structures of TNF-α and IL-6 were downloaded from the Protein Data Bank (PDB, https://www.rcsb.org/). All receptors were removed the water molecules and added the hydrogen atoms, which were conversed to PDBQT format via AutoDock software. And then the optimal conformation was found to obtain the docking structure with the lowest energy. Finally, the outcomes of the docking process were performed for a comprehensive visualization using PyMOL software (version 2.2, https://PyMOl.org/2/) [18].

### Animal and experimental design

Forty male C57BL/6 J mice (weight, 18–20 g, 4-week-age) were purchased from Sibeifu (Beijing) Biotechnology Co., Ltd. (Animal license NO. SCXK(Jing)-2019-0010). The mice were maintained in an environmentally controlled space at 25 ± 1 °C, 60 ± 5% relative humidity, and a 12 light-12 dark cycle, with unlimited access to food and water. The experiments were approved by the Animal Ethics Committee of Changchun University of Chinese Medicine and Institutional Animal Care (Approval No. 2024250).

After acclimatization for 1 week, 10 randomly selected mice were divided into Control group that were intraperitoneally injected with normal saline (NS); 30 mice were fasted for 12 h and then intraperitoneally injected with STZ (50 mg/kg) for five days to establish diabetic mice. One week after the last model was established, random blood glucose (RBG) was detected in diabetic mice. A diabetic model was successfully established in mice with RBG ≥ 11.1 mmol/L. 30 Diabetic mice were randomized into three groups (*n* = 10 /group): Model group (STZ), CaD group (STZ + Calcium dobesilate capsules, 0.2 g/kg), and YQWYF group (STZ + YQWYF, 1.0 g/kg) for 18 weeks. The other groups were administered equal volumes of normal saline. The dosage of YQWYF in the mice was calculated based on using the body surface area (BSA) normalization method from human to animal [19, 20] and the results of previous studies [8, 11]. All mice were then euthanized by cervical dislocation under anesthesia with 30 mg/kg pentobarbital sodium (PS) by intraperitoneal injection after fasting for 12 h. No mice showed abnormal signs or reached humane endpoints throughout the experiment.

### Biochemical measurements of urine and serum

The mice were placed in metabolic cages to collect 24-h urine at the 9th and 18th weeks. Glu (u-Glu, cat. no. 020586), Crea (u-Crea, cat. no. 020587), MAlb (u-MAlb, cat. no. 024898), CysC (u-CysC, cat. no. 024897) and UA (u-UA, cat. no. 024896) were detected with biochemical reagent kits (Mindray, Shenzhen, China) via a BS-240VET automatic biochemical analyzer (Mindray). The mice were fasted for 12 h, and then, blood from the ophthalmic vein was collected under anesthesia (30 mg/kg PS, concentration 5 mg/ml, dose 60 µl/10 mg body weight)) to detect Glu (b-Glu, cat. no. 020586), Crea (b-Crea, cat. no. 020587), Urea (b-Urea, cat. no. 020583), Hcy (b-Hcy, cat. no. 024882), high density lipoprotein-cholesterol (HDL-C, cat. no. 025018), low density lipoprotein-cholesterol (LDL-C, cat. no. 025025) and total cholesterol (TC, cat. no. 020588) using biochemical reagent kits (Mindray).

### Inflammatory cytokines and oxidative stress biomarkers

Serum interleukin (IL)-1β (cat. no. JM-02323M1), IL-6 (cat. no. JM-02446M1), and tumor necrosis factor (TNF)-α (cat. no. JM-02415M1) were measured by commercial ELISA kits (Jingmei Biotechnology, Jiangsu, China). Glutathione (GSH, cat. no. JM-02941M1), glutathione peroxidase (GPx, cat. no. JM-03038M1), catalase (CAT, cat. no. J2833-A), superoxide dismutase (SOD, cat. no. J2389-A), malondialdehyde (MDA, cat. no. J9264-A), lactate dehydrogenase (LDH, cat. no. JM-11330M1), and reactive oxygen species (ROS, cat. no. J30360-A) in the kidney were detected via ELISA kits (Jingmei Biotechnology, Jiangsu, China).

### Histological study

Fresh kidneys were fixed with 4% paraformaldehyde, and paraffin-embedded kidney sections were prepared for hematoxylin–eosin (HE) staining, periodic acid-Schiff (PAS) staining, and Masson’s trichrome (Masson) staining. Images were captured using AxioScan 7 (Carl Zeiss AG, Germany).

### Fecal 16S rRNA gene sequencing

Fresh feces from each mouse were collected at the 18th week and stored at -80 °C. 16S rRNA gene sequencing was performed by Novogene Co., Ltd. (Beijing, China). Briefly, total DNA from a fecal sample (100 ~ 150 mg) was extracted via a QIAamp DNA Stool Mini Kit (Qiagen, Germany). The V3-V4 sequenced region of the gene was amplified with a universal primer (forward: 5’-CCTAYGGGRBGCASCAG-3’ and reverse: 5’-GGACTACNNGGGTATCTAAT-3’). The PCR products were purified via magnetic beads and then detected on a 2% agarose gel, after which the target band was collected. The sequencing library was prepared and evaluated via a Qubit instrument and real-time PCR for quantitative analysis, and size distribution was detected via a bioanalyzer. The quality-quality libraries were sequenced via Illumina platforms. The sequences were assigned to amplicon sequence variants (ASVs) at 97% similarity. The Majorbio Cloud Platform (https://magic.novogene.com/) was used for bioinformatics analysis [21].

### Fecal bile acid profiling analysis by untargeted metabolite analysis

Fresh feces from each mouse (100 mg) were pulverized with liquid nitrogen, and then the homogenates were resuspended in precooled 80% methanol. The mixture was subsequently centrifuged at 15,000 × *g* for 20 min at 4 °C. A portion of the supernatant was diluted with water to obtain a final concentration of 53% methanol. The supernatant was subsequently injected into a UHPLC-MS/MS system (Thermo Fisher, Germany) coupled with an Orbitrap Q ExactiveTM HF mass spectrometer (Thermo Fisher, Germany) on a Hypersil Gold column (100 mm × 2.1 mm, 1.9 μm) with a flow rate of 0.2 mL/min. The mobile phase included methanol (A) and 0.1% formic acid (B). The gradient elution conditions were as follows: 2% A, 1.5 min; 2–85% A, 3 min; 85–100% A, 10 min;100–2% A, 10.1 min; 2% A, 12 min. For both positive and negative polarity modes, a Q ExactiveTM HF mass spectrometer was used with the following parameters: spray voltage, 3.5 kV; capillary temperature, 320 °C; sheath gas flow rate, 35 psi; aux gas flow rate, 10 L/min; S-lens RF level, 60; and aux gas heater temperature, 350 °C [22].

The raw data files generated by UHPLC-MS/MS were processed using the Compound Discoverer 3.3 (CD3.3, ThermoFisher) to perform peak alignment, peak picking, and quantitation for each metabolite. The main parameters were set as follows: peak area was corrected with the first QC, actual mass tolerance, 5 ppm; signal intensity tolerance, 30%; and minimum intensity, et al. After that, peak intensities were normalized to the total spectral intensity. The normalized data was used to predict the molecular formula based on additive ions, molecular ion peaks and fragment ions. And compounds whose CVs of relative peak areas in QC samples were greater than 30% were removed, and finally the metabolites' identification and relative quantification results were obtained. We applied univariate analysis (t-test) to calculate the statistical significance (*P*-value). The metabolites with VIP > 1 and *P* < 0.05 and fold change ≥ 2 or FC ≤ 0.5 were considered to be differential metabolites. Volcano plots were used to filter metabolites of interest which based on log2 (FoldChange) and -log10 (*P*-value) of metabolites by ggplot2 in R language. These metabolites were annotated using the KEGG database (https://www.genome.jp/kegg/pathway.html).

### Real-time quantitative PCR (RT-qPCR)

The total RNA from kidney was prepared using TRIzol reagent. RT qPCR was conducted by reverse transcription using FastKing gDNA Dispelling RT SuperMix (TIANGEN BIOTECH CO., LTD., Beijing, China). The sequences of the primers used were as follows: β-actin (Forward, GATGGTGGGAATGGGTCAGAAGG, Reverse, TTGTAGAAGGTGTGGTGCCAGATC), IL-1β (Forward, TTCAGGCAGG CAGTATCACTC, Reverse, GAAGGTCCACGGGAAAGACAC), IL-6 (Forward, GA CTGATGCTGGTGACAACC, Reverse, AGACAGGTCTGTTGGGAGTG), TNF-α (Forward, ACGCTCTTCTGTCTACTGAACTTCG, Reverse, TGGTTTGTGAGTGT GAGGGTCTG), NF-κB (Forward, ATGGGAAACCGTATGAGCCTGTG, Reverse, AGTTGT AGCCTCGTGTCTTCTGTC), Claudin-1 (Forward, GTGTCC TACTTTCCTGCTCCTGTC, Reverse, AGAAGGTGTTGGCTTGGGATAAGG), ICAM-1 (Forward, GGAGACGCAGAGGAC CTTAACAG, Reverse, GGCTTCACACTTCACAGTTACTTGG), RAGE (Forward, AGCCACT GGAATTG TCGATGAGG, Reverse, ATCTGGTAGACTCGGACTCGGTAG), Zo-1 (Forward, ACCCGAAACTG ATGCTGTGGATAG, Reverse, GCTGGCTGGCTGTACTGTGA G). Relative gene expression was calculated using the 2^−△△Ct^ method with *β*-actin normalization.

### Western blot detection

Western blot was performed as previously reported [17]. Briefly, proteins from the kidney were extracted with lysis buffer. A total of 35 μg of protein was loaded and separated via sodium dodecyl sulfate‒polyacrylamide gel electrophoresis (SDS-PAGE) and then transferred to a PVDF membrane. The membranes were incubated with primary antibodies, including anti-β-actin (cat. no. 66009–1-Ig, Proteintech, China), anti-IL-1β (cat. no. HY-P80720, MCE, America), anti-IL-6 (cat. no. HY-P80189A, MCE), anti-TNF-α (cat. no. 17590-1-AP, Proteintech), anti-receptor for advanced glycation endproduct (RAGE, cat. no. 83759-5-RR, Proteintech), anti-intercellular adhesion molecule 1 (ICAM-1, cat. no. BS2980, Bioworld technology, America), anti-claudin-1 (cat. no. BS40537, Bioworld technology), anti-CYP7A1 (cat. no. SC-518007, Santa Cruz Biotechnology, Bolivia), anti-CYP8B1 (cat. no. ab191910, Abcam, Britain), and anti-farnesoid X receptor (FXR, cat. no. #72105, Cell signaling technology, America), anti-NF-κB p65 (cat. no. 10745-1-AP, Proteintech), and anti-p-NF-κB p65 (cat. no. BZ16389, Bioworld technology) followed by incubation with secondary antibodies for 2 h. The bands were visualized with a FlourChem HD2 system. The relative protein expression was normalized to that of β-actin.

### Statistical analysis

The data were analyzed by GraphPad Prism 8.0 software. After conducting normality tests (Shapiro–Wilk test) and homogeneity of variance tests (Brown-Forsythe's test), data conforming to a normal distribution were analyzed by one-way ANOVA. Data conforming to a normal distribution were analyzed by one-way ANOVA. For datasets with homogeneous variance, pairwise comparisons were performed using the LSD method, while Dunnett’s T3 test was applied for datasets with heterogeneous variance. Results were expressed as mean ± standard error of the mean (SEM). Pearson correlation analysis was used to calculate correlation coefficients for normally distributed data. The data of gut microbiota and bile acids were analyzed using Student’s t test. *P* < 0.05 was considered to indicate statistical significance.

## Results

### Chemical ingredients of YQWYF

**Fig. 1:**
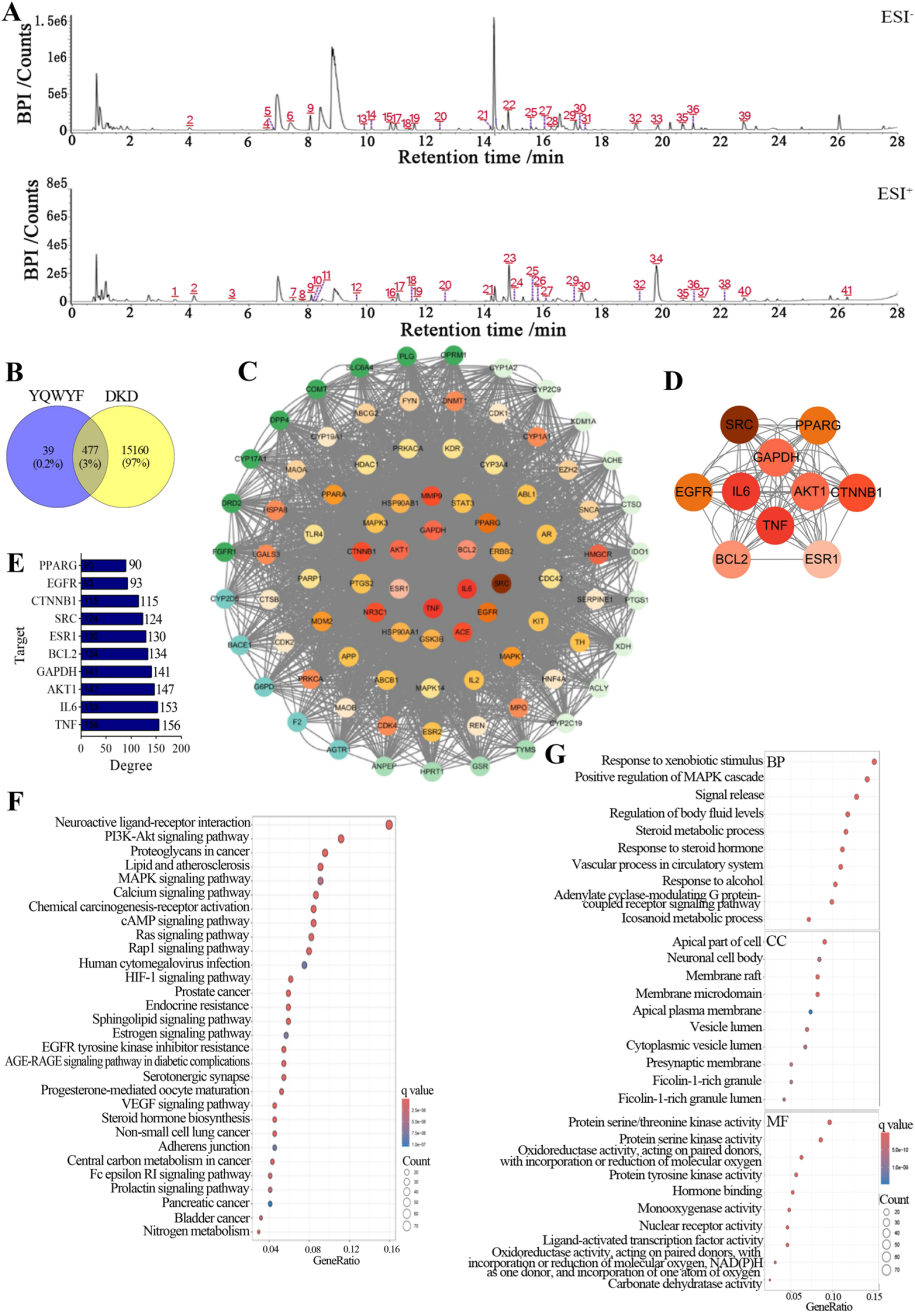
Function enrichment analysis of 33 compounds from YQWYF treatment targets in DKD. **A** 41 Chemical ingredients of YQWYF in negative (ESI-) and positive (ESI +) ion modes. **B** Venn diagram of the therapeutic targets of 33 compounds in YQWYF and the potential targets for DKD. **C** Protein–protein interaction (PPI) network of 477 targets. **D** PPI network of the top 10 targets. A darker color represents a higher degree value. **E** Degree values of top 10 targets. **F** Top 30 KEGG pathway enrichment analysis of 477 targets. **G** Gene Ontology (GO) of 477 targets enrichment analysis. BP, biological process; CC, cellular component; MF, molecular function

Figure 1A showed the base peak chromatograms of YQWYF using UPLC-Q-TOF/MS. A total of 41 compounds from Astragali Radix (AR), Lycii Fructus (LF), Eriocauli Flos (EF), Cinnamomi Ramulus (CR), and Typhae Pollen (TP) in YQWYF were confirmed according to UNIFI software and reference standards (supplementary Table S1). YQWYF mainly included flavones, glycosides, and a few amides and fatty acids. Rhamnocitrin, quercetin-3-O-rutinoside-(1–2)-O-rhamnoside, typhaneoside, calycosin-7-O-β-D-glucoside, kaempferol-3-O-neohesperidoside, isorhamnetin-3-O-neohespeidoside, narcissoside, ononin, astraisoflavan-7-O-β-D-glucoside, calycosin, jaranol, naringenin, kaempferol, isorhamnetin, and formononetin belonged to flavones; markhamioside F, asperulosidic acid, astragaloside VII, astragaloside IV, astragaloside III, astragaloside II, isoastragaloside II, soyasaponin I, astragaloside I, and isoastragaloside I belonged to glycosides. A total of 21, 9 and 6 unique ingredients were identified in AR, TP and LF, respectively. 12-Phenyldodecanoic acid, 9-hydroxy-10,12-octadecadienoic acid and methyl ricinolate were identified from AR and TP; 16-meprednisone acetate was identified from CR and TP; and narcissoside was identified from TP and LF.

### Potential targets of YQWYF for DKD treatment were predicted using network pharmacology

Among the 516 targets associated with 33 compounds of YQWYF and 15,637 targets associated with DKD, 477 common targets between YQWYF and DKD were identified (Fig. 1B). The topological parameters of the network were revealed in 241 nodes with 3122 edges (Fig. 1C). The top 10 core targets of YQWYF for DKD treatment included mainly TNF, IL6, AKT1, GAPDH, BCL2, ESR1, SRC, CTNNB1, EGFR, and PPARG (Fig. 1D), and the targets ranking and topological parameters of top 10 targets was showed in Fig. 1E and Table 1. In particular, TNF and IL6 among the 10 core targets were important targets of YQWYF for DKD treatment. In the top 30 KEGG pathway enrichment analysis (Fig. 1F), the major enrichment pathways included ‘PI3K-Akt signaling pathway’, ‘MAPK signaling pathway’, and ‘AGE-RAGE signaling pathway in diabetic complications’. GO enrichment analysis revealed that 477 proteins were involved in ‘positive regulation of MAPK cascade’, ‘steroid metabolic process’, and ‘response to steroid hormone’ in the biological process (BP), participated in ‘apical part of cell’, ‘neuronal cell body’, ‘membrane microdomain’ in the cellular component (CC), and related to ‘protein serine/threonine kinase activity’, ‘hormone binding’, ‘nuclear receptor activity’ in molecular function (MF, Fig. 1G).

**Table 1:** Intersection targets with the top 10 degree

Target	Degree	Betweenness	Closeness
TNF	156	0.026041111	0.972225711
IL6	153	0.026040003	0.959253251
AKT1	147	0.024013333	0.897566323
GAPDH	141	0.024802222	0.860472251
BCL2	134	0.023690147	0.826111111
ESR1	130	0.022130999	0.785400325
SRC	124	0.018927771	0.770899323
CTNNB1	115	0.015332511	0.755788888
EGFR	93	0.013888888	0.729014729
PPARG	90	0.012152777	0.700089125

**Fig. 2:**
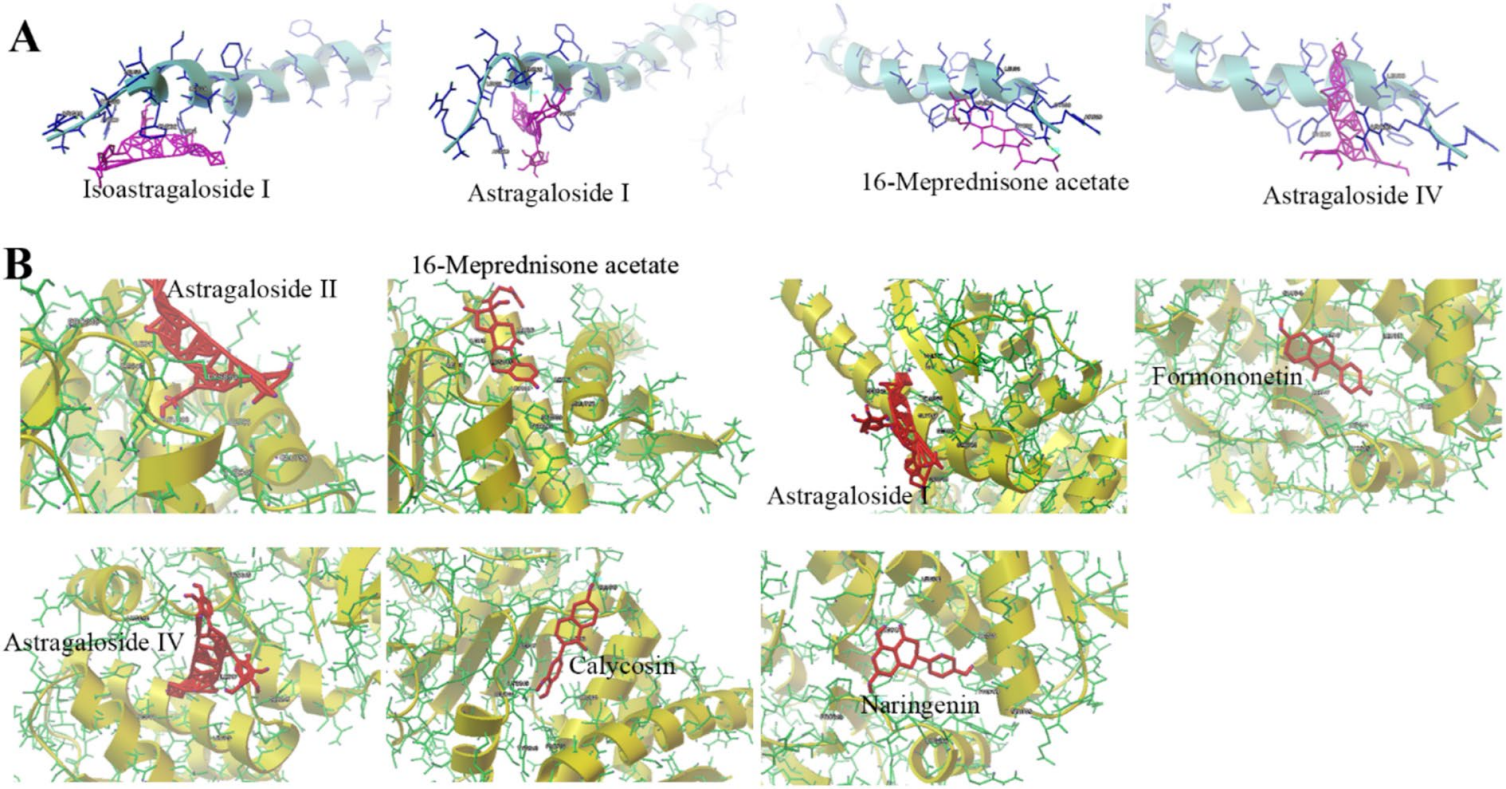
Molecular docking of DKD-related targets (TNF-α and IL-6) with the main compounds of YQWYF. **A** Molecular models of the binding of isoastragaloside I, astragaloside I, 16-meprednisone acetate and astragaloside IV with TNF-α. **B** Molecular models of the binding of astragaloside II, 16-meprednisone acetate, astragaloside I, formononetin, astragaloside IV, calycosin, and naringenin with IL-6

We docked 2 key targets (TNF-α and IL-6) with 33 compounds in YQWYF to confirm compound-target interactions. A binding affinity of less than − 5.0 kcal/mol indicated good interactions. Table 2 showed that isoastragaloside I, astragaloside I, 16-meprednisone acetate, and astragaloside IV showed the strong affinity with TNF-α, the binding energy was − 7.08, − 6.82, − 6.51, − 5.58 kcal/mol, respectively. Astragaloside II, 16-meprednisone acetate, astragaloside I, formononetin, astragaloside IV, calycosin and naringenin showed the strong affinity with IL-6, the binding energy was − 6.84, − 6.83, − 6.6, − 5.78, − 5.54, − 5.14 and − 5 kcal/mol, respectively. Figure 2 showed the conformations of compounds with key targets (TNF-α and IL-6). These results indicated that three components of YQWYF (astragaloside I, 16-meprednisone acetate, and astragaloside IV) showed excellent anti-inflammatory effects.

**Table 2:** The results of molecular docking

Compounds	Binding energy (kcal/mol)		Compounds	Binding energy (kcal/mol)	
	TNF-α	IL-6		TNF-α	IL-6
Isoastragaloside I	− 7.08	− 3.13	L-Phenylalanine	− 3.92	− 4.17
Astragaloside I	− 6.82	− 6.60	*p*-Coumaric acid	− 3.85	− 4.45
16-Meprednisone acetate	− 6.51	− 6.83	Typhaneoside	− 3.81	− 0.44
Astragaloside IV	− 5.58	− 5.54	Isoastragaloside II	− 3.76	− 4.4
Ononin	− 4.84	− 4.93	Adenosine	− 3.67	− 4.1
Formononetin	− 4.73	− 5.78	Guanosine	− 3.59	− 4.33
Naringenin	− 4.71	− 5.00	12-Phenyldodecanoic acid	− 3.53	− 3.66
Indolelactic acid	− 4.62	− 4.60	Methyl ricinolate	− 3.34	− 3.37
Calycosin	− 4.48	− 5.14	9-Hydroxy-10,12-octadecadienoic acid	− 3.15	− 3.61
Astragaloside III	− 4.39	− 3.7	Linoleic acid	− 3.05	− 3.33
Kaempferol	− 4.30	− 4.83	Soyasaponin I	− 2.78	− 2.34
Jaranol	− 4.28	− 4.66	Asperulosidic acid	− 2.29	− 3.43
Astragaloside II	− 4.27	− 6.84	Kaempferol-3-*O*-neohesperidoside	− 1.92	− 2.73
Rhamnocitrin	− 4.26	− 4.94	Quercetin-3-*O*-rutinoside-(1–2)-*O*-rhamnoside	− 1.6	− 1.54
Astragaloside VII	− 3.99	–	Isorhamnetin-3-*O*-neohespeidoside	− 1.4	− 1.41
Isorhamnetin	− 3.95	− 4.36	Narcissoside	− 1.36	− 1.58
Scopoletin	− 3.94	− 4.63

### YQWYF improved the biochemical indicators of urine and serum in DKD mice

**Fig. 3:**
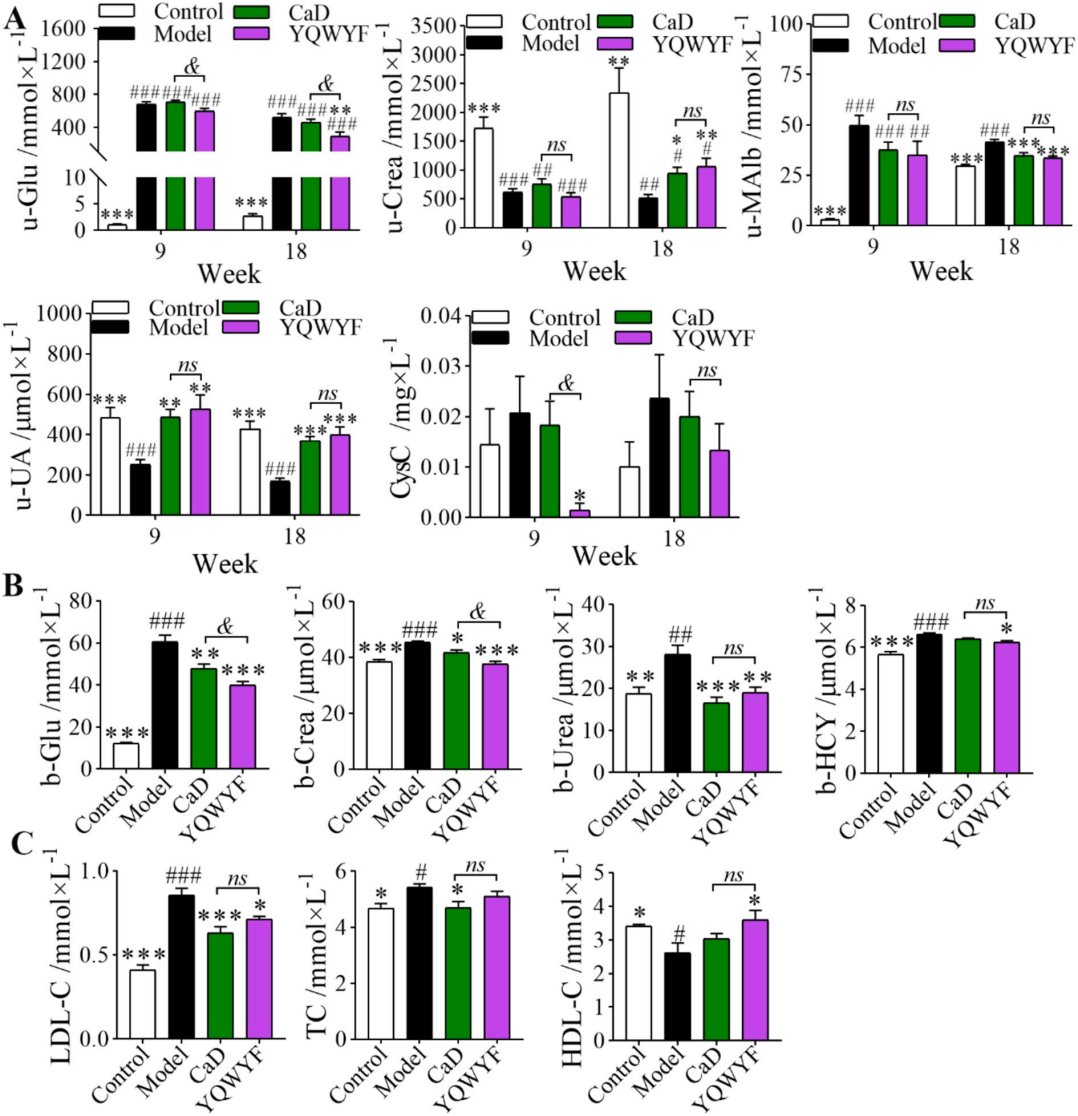
YQWYF improved the biochemical indicators of urine and serum in DKD mice. **A** The biochemical indicators of urine at the 9th and 18th weeks (*n* = 10). **B** Serum biochemical indicators of serum at the 18th week (*n* = 10). **C** Lipid metabolism indexes of the serum at the 18th week (*n* = 10). The data are expressed as mean ± SEM, ^*^P < 0.05, ^**^P < 0.01, and ^***^P < 0.001 compared to Model group; ^#^*P* < 0.05, ^##^*P* < 0.01, and ^###^*P* < 0.001 compared to Control group; ^&^*P* < 0.05 compared to CaD group; *ns* showed no significance between two groups

Glu, Crea, MAlb, UA, and CysC levels of urine in the mice were detected at the 9th and 18th weeks. Figure 3A showed that STZ caused the high-Glu, high-MAlb, low-Crea and low-UA of urine in DKD mice at the 9th and 18th weeks (*P* < 0.01). In the drug administration group, YQWYF significantly elevated the u-UA level of DKD mice at the 9th and 18th weeks (*P* < 0.01). YQWYF had almost difficult to improve the u-Glu, u-Crea and u-MAlb levels of the model mice at the 9th week (*P* > 0.05); but YQWYF obviously decreased the u-Glu and u-MAlb levels and increased the u-Crea level of the mice compared to Model group at the 18th week (*P* < 0.01). YQWYF also reduced the CysC level in DKD mice; however, the difference was not statistically significant at the 18th week (*P* > 0.05). In the biochemical indicators of serum, Glu, Crea, Urea and Hcy in DKD mice obviously increased compared to Control group (Fig. 3B, P < 0.01). After 18 weeks of drug administration, YQWYF obviously decreased the levels of b-Glu, b-Crea, b-Urea and b-Hcy in DKD mice (*P* < 0.05). Importantly, YQWYF reduced obviously the levels of u-Glu, b-Glu, and b-Crea compared to CaD group (*P* < 0.05). These results showed that YQWYF reduced blood glucose and improved the biochemical indicators of kidney in DKD mice.

In the lipid metabolism indices of the serum, LDL-C and TC levels obviously increased, and HDL-C level decreased in DKD mice induced by STZ (Fig. 3C, P < 0.05). YQWYF obviously improved LDL-C and HDL-C levels in DKD mice (*P* < 0.05); however, the serum TC levels of the mice in YQWYF group were lower than that in Model group; however, the differences between Model and YQWYF groups were not statistically significant (*P* > 0.05). CaD significantly reduced the levels of LDL-C and TC (*P* < 0.05). Thus, supplementation with YQWYF improved lipid metabolism abnormalities in DKD mice.

### YQWYF improved inflammatory cytokine levels in the serum and kidneys of DKD mice

**Fig. 4:**
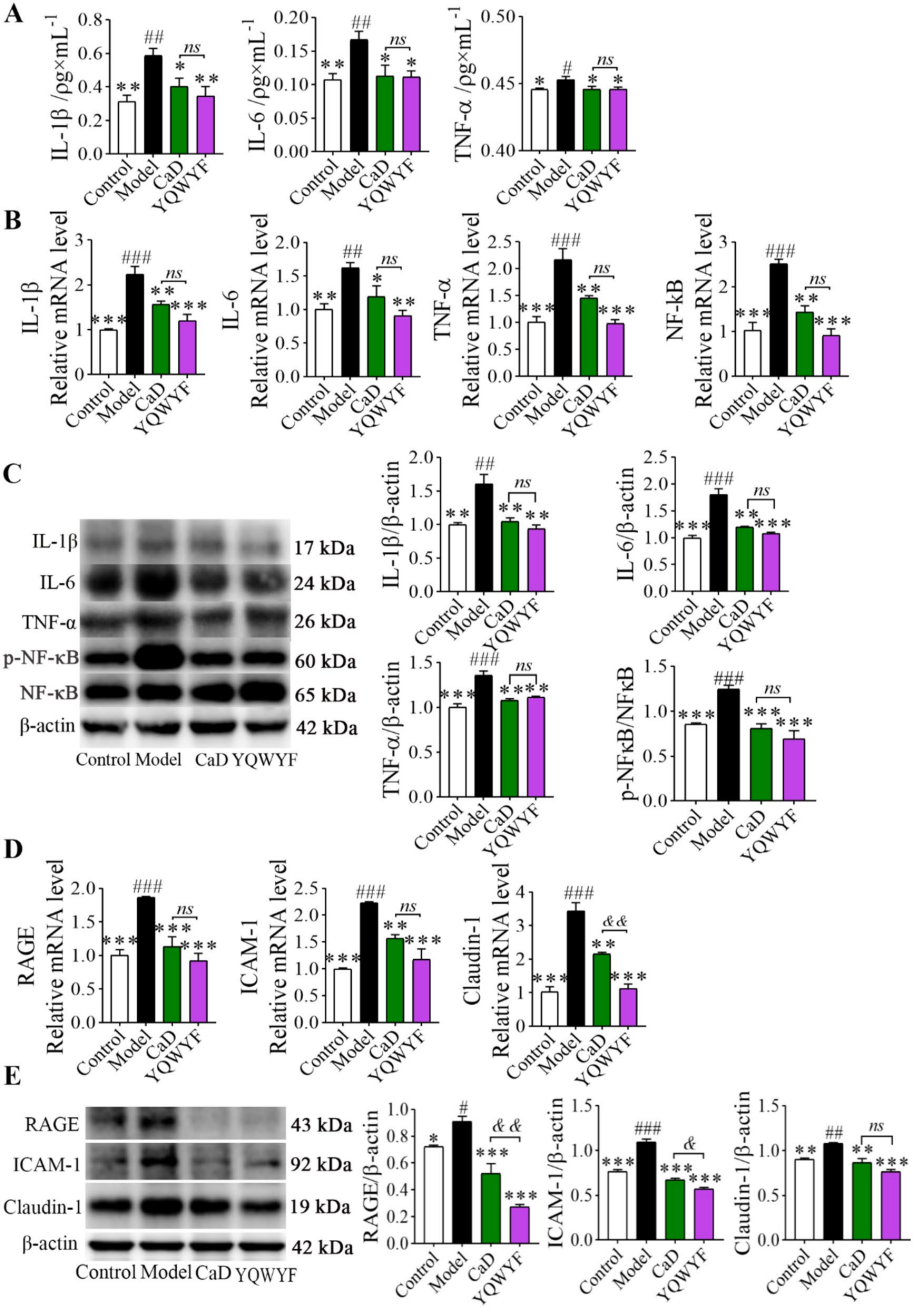
YQWYF improved the levels of inflammatory cytokines in serum and kidney, and renal tissue factors in DKD mice. **A** The inflammatory cytokines in mouse serum (*n* = 10). **B** The mRNA levels of inflammatory cytokines in the kidney (*n* = 6). **C** The protein levels of inflammatory cytokines in the kidney (*n* = 4–6). **D** The mRNA levels of renal tissue factor (*n* = 6). **E** The protein levels of renal tissue factor in kidney (*n* = 3). The data are expressed as mean ± SEM, ^*^P < 0.05, ^**^P < 0.01, and ^***^P < 0.001 compared to Model group; ^#^*P* < 0.05, ^##^*P* < 0.01, and ^###^*P* < 0.001 compared to Control group. ^&^*P* < 0.05, and ^&&^*P* < 0.01 compared to CaD group; *ns* showed no significance between two groups

The levels of the inflammatory cytokines IL-1β, IL-6, and TNF-α in the serum (Fig. 4A) and kidneys (Fig. 4B and 4C) of DKD mice were higher than those in Control group (*P* < 0.05); moreover, the NF-κB mRNA level in DKD mice was obviously greater than that in Control group (Fig. 4B, P < 0.001). YQWYF and CaD obviously decreased the levels of IL-1β, IL-6, and TNF-α in the serum and kidneys of model mice (Fig. 4A–C, P < 0.05), and inhibited the level of the NF-κB (Fig. 4B, P < 0.001) and activation of p-NF-κB (Fig. 4C, P < 0.001). The level of these inflammatory cytokines between YQWYF and CaD groups had no statistics difference (*P* > 0.05). Therefore, both YQWYF and CaD inhibited the release and expression of inflammatory cytokines in DKD mice.

Furthermore, we detected kidney tissue factors. Figure 4D and 4E showed that long-term exposure to high glucose in the kidney caused an increase in RAGE, ICAM-1, and Claudin-1 mRNA and protein levels (*P* < 0.05). YQWYF and CaD obviously reduced the expressions of RAGE, ICAM-1, and claudin-1 in the kidney compared with those in Model group (Fig. 4D and 4E, *P* < 0.01). Especially, the inhibition of YQWYF on RAGE and ICAM was stronger than CaD (4E, *P* < 0.05). These results indicated that YQWYF could reduce the indices related to kidney injury in DKD mice.

### YQWYF improved kidney injury in DKD mice

**Fig. 5:**
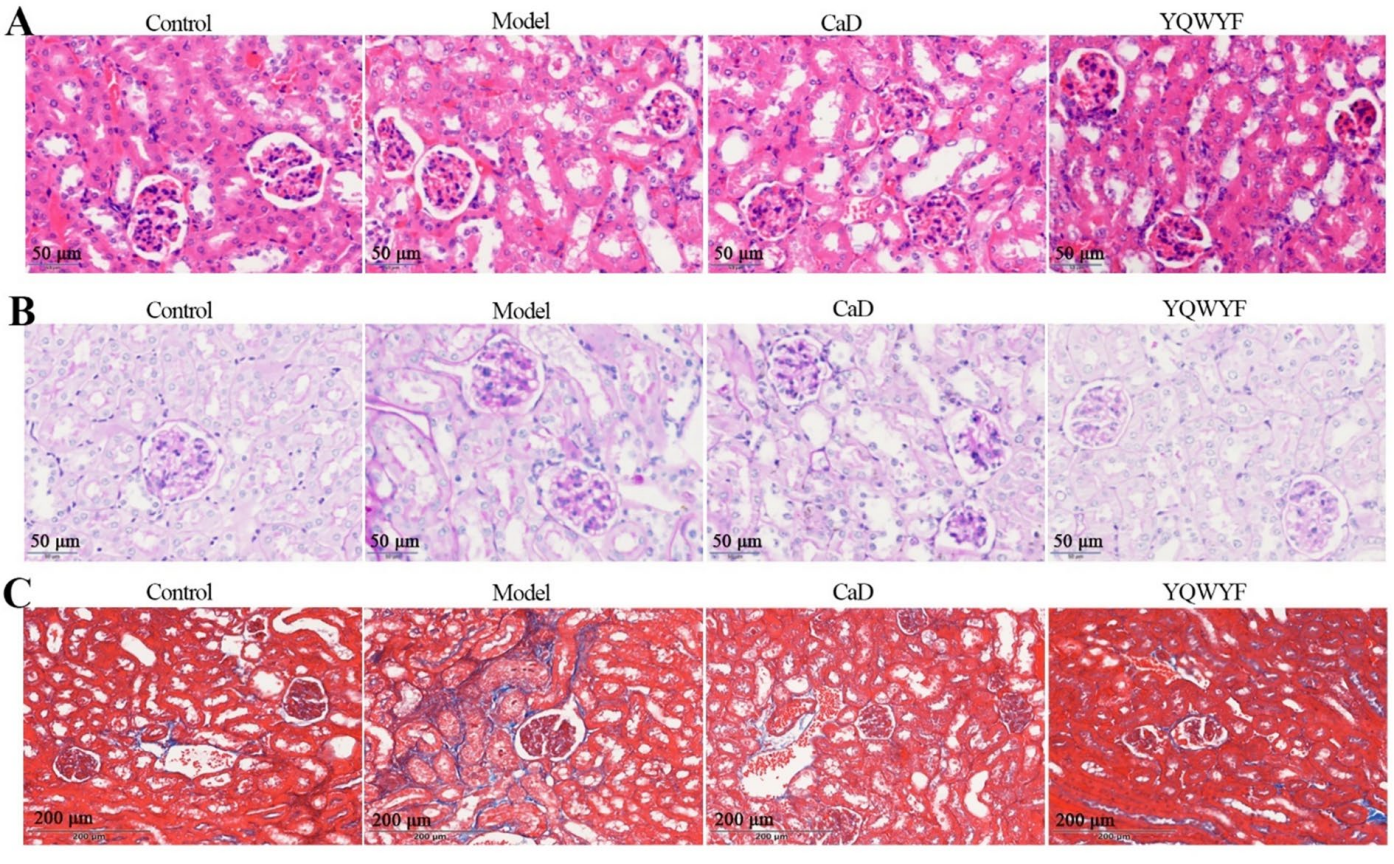
YQWYF improved kidney injury in DKD mice. **A** HE staining of kidney tissue. **B** PAS staining of kidney tissue. **C** Masson staining of kidney tissue

Renal sections were stained with HE, PAS and Masson. Figure 5A–C showed that the morphology of the glomerular and renal tubules was normal, without hyperplasia or other lesions in Control group. HE and PAS staining demonstrated that the glomerular volume was obviously increased; the tubular epithelial cells were edematous, the cytoplasm was loosely stained, and the nuclei were pyknotic and hyperstained in Model group (Fig. 5A and 5B). In addition, Masson staining revealed STZ-induced renal collagen deposits and fibrosis (Fig. 5C). YQWYF improved pathological changes in the renal microstructure, such as a reduction in blister degeneration, necrotic cells and collagen deposition, and the cells were arranged more closely and orderly.

### YQWYF improved oxidative stress biomarkers in the kidneys of DKD mice

**Fig. 6:**
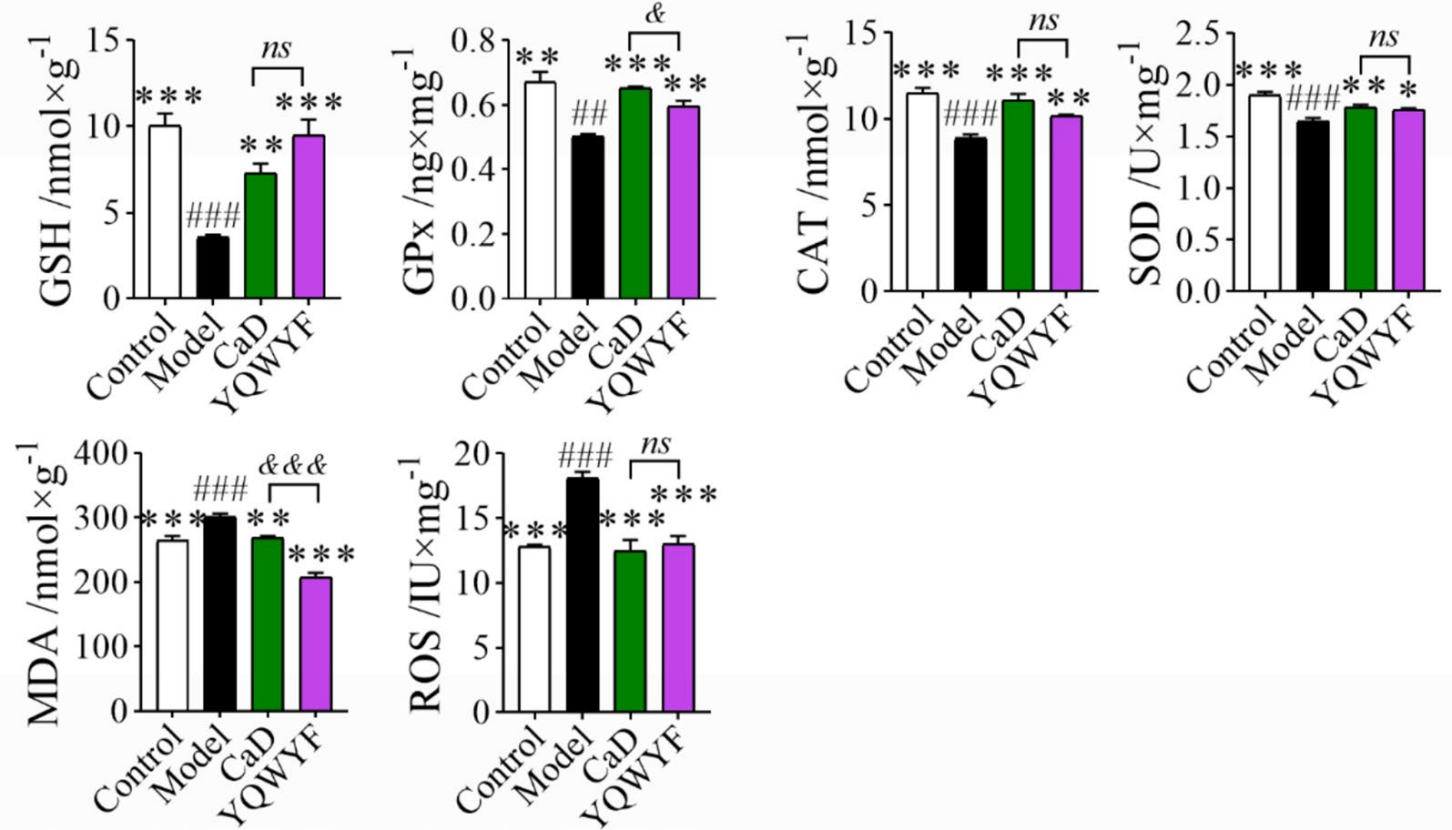
YQWYF improved oxidative stress biomarkers of kidney in DKD mice. The data are expressed as mean ± SEM (*n* = 10), ^*^P < 0.05, ^**^P < 0.01, and ^***^P < 0.001 compared to Model group; ^##^*P* < 0.01, and ^###^*P* < 0.001 compared to Control group. ^&&&^*P* < 0.001 compared to CaD group; *ns* showed no significance between two groups

High glucose causes oxidative stress damage in the kidney. Figure 6 showed that the levels of GSH, GPx, CAT, and SOD of kidney in DKD mice were lower than those in Control group (*P* < 0.01), and the levels of MDA and ROS were increased (*P* < 0.001). YQWYF and CaD obviously increased the GSH, GPx, CAT, and SOD levels in the kidney and decreased the MDA and ROS levels (*P* < 0.05). Meanwhile, YQWYF reduced significantly the MDA content compared to CaD (*P* < 0.001). These results showed that YQWYF inhibited oxidative stress damage in DKD mice induced by STZ.

### YQWYF improved the relative abundance of the gut microbiota in DKD mice

**Fig. 7:**
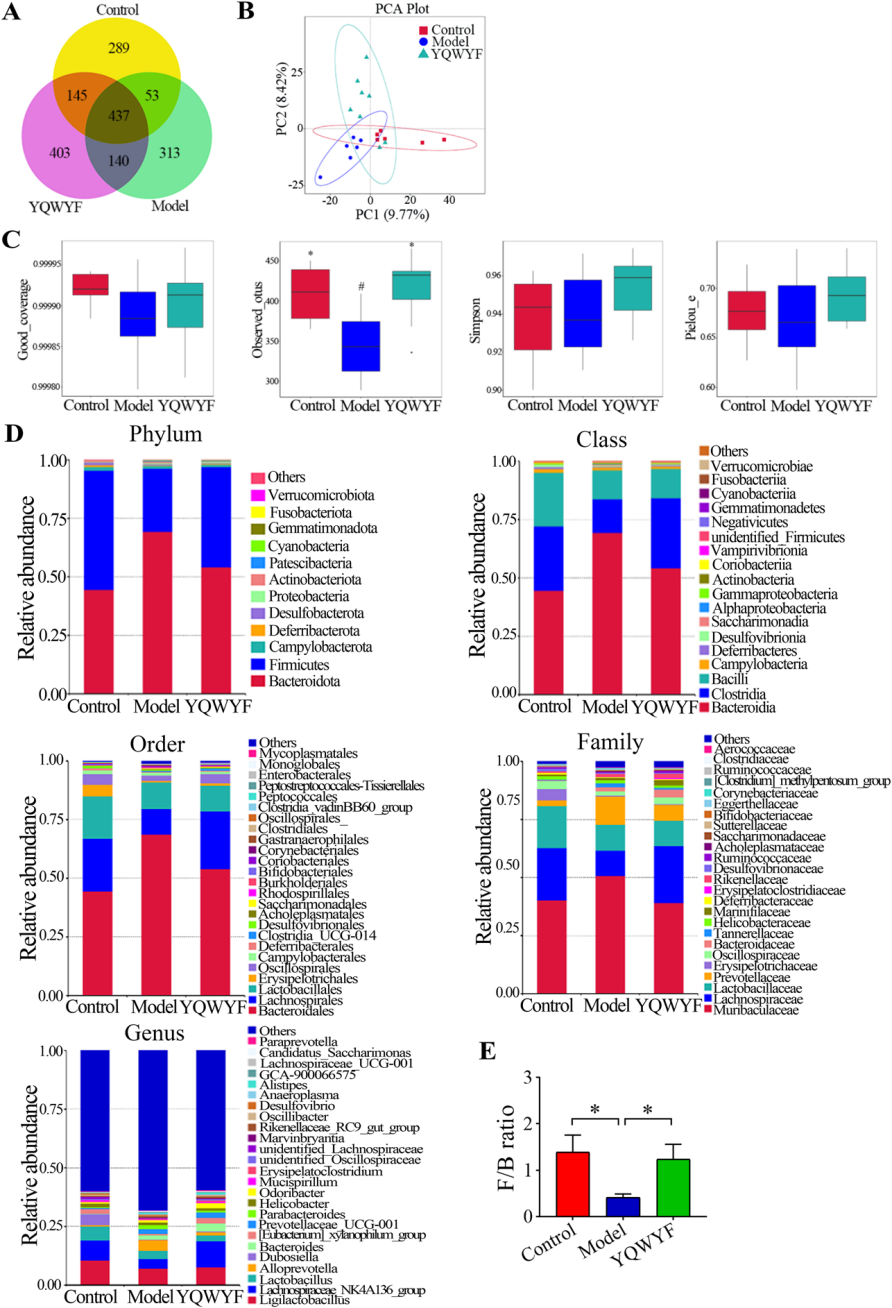
YQWYF improved the relative abundance of the gut microbiota in DKD mice. **A** Venn diagram of overlap ASVs among groups (*n* = 6–8). **B** principal component analysis (PCA, *n* = 6–8). **C** Alpha-diversity estimators by Good_coverage, observed_otus, simpson, and pielou_e indexes (*n* = 6–8). **D** The relative abundances of bacterial taxonomic profiling at the phylum (top 12), class (top 18), order (top 25), family (top 25), genus (top 25) levels. **E** *Firmicutes*/*Bacteroidota* (F/B) ratio (*n* = 6–8). The data are expressed as mean ± SEM, ^*^P < 0.05 compared to Model group; ^#^*P* < 0.05 compared to Control group

There were 289, 313 and 403 unique amplicon sequence variants (ASVs) in Control, Model, and YQWYF groups, respectively (Fig. 7A). Principal component analysis (PCA) showed the differences of samples among Control, Model, and YQWYF groups (Fig. 7B). The Good_coverage index reflected the sequencing coverage rate; the observed_otus index reflected richness; the Simpson index reflected dominant species; and the Pielou_e index reflected evenness. Figure 7C showed that the good_coverage, observed_otus, Simpson, and Pielou_e indexes of DKD mice in Model group were lower than those in Control group; specifically, the observed_otus index decreased significantly in Model group (*P* < 0.05). However, four indexes were increased in model mice treated with YQWYF, and the observed_otus had significant difference (*P* < 0.05). These results showed that YQWYF could increase the species, richness and evenness of the gut microbiota in DKD mice. Figure 7D showed the relative abundances of bacterial taxonomic profiling among Control, Model and YQWYF groups at the phylum (top 12), class (top 18), order (top 25), family (top 25), and genus (top 25) levels; obviously, YQWYF recovered the relative abundance of the gut microbiota at different levels compared with Model group. At the phylum level, the *Firmicutes/Bacteroides* (F/B) ratio in Model group was lower than that in Control group, indicating a gut microbiota disorder; however, YQWYF obviously increased the F/B ratio (Fig. 7E, P < 0.05) and recovered the relative abundances of the abnormal microbiota.

**Fig. 8:**
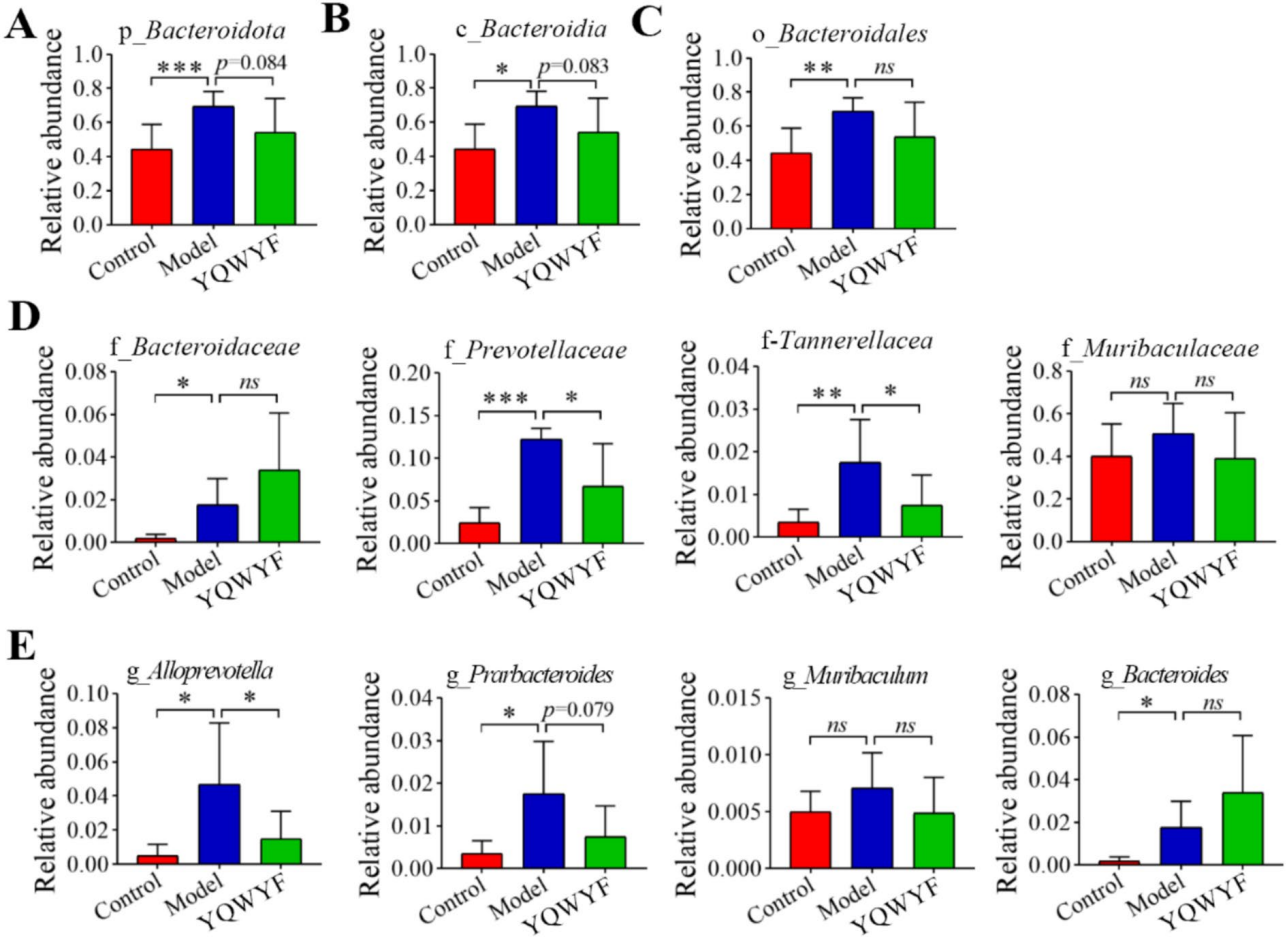
YQWYF improved the relative abundance of *bacteroidota* in different levels in DKD mice. **A** The relative abundance of *bacteroidota* in phylum level (*n* = 6–8). **B** The relative abundance of *bacteroidia* in class level (*n* = 6–8). **C** The relative abundance of *bacteroidales* in order level (*n* = 6–8). **D** The relative abundance of *bacteroidaceae* in family level (*n* = 6–8). **E** The relative abundance of *bacteroides* in genus level (*n* = 6–8). The data are expressed as mean ± SEM, ^*^P < 0.05, ^**^P < 0.01 and ^***^P < 0.001 compared to Model group; *ns* showed no significance between two groups

**Fig. 9:**
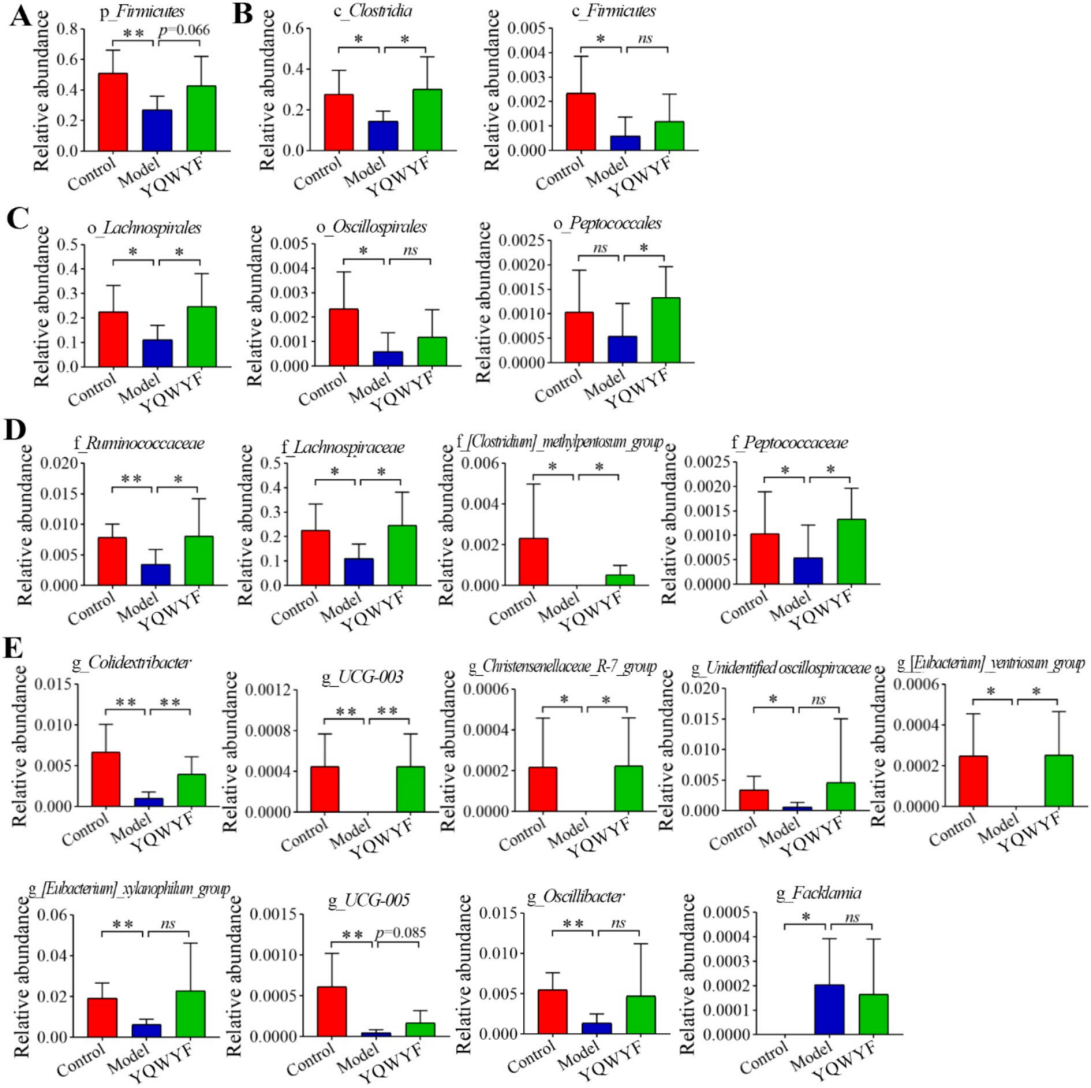
YQWYF improved the relative abundance of *firmicutes* in different levels in DKD mice. **A** The relative abundance of *firmicutes* in phylum level (*n* = 6–8). **B** The relative abundance of *firmicutes* in class level (*n* = 6–8). **C** The relative abundance of *firmicutes* in order level (*n* = 6–8). **D** The relative abundance of *firmicutes* in family level (*n* = 6–8). **E** The relative abundance of *firmicutes* in genus level (*n* = 6–8). The data are expressed as mean ± SEM, ^*^P < 0.05, and ^**^P < 0.01 compared to Model group; *ns* showed no significance between two groups

In the differential analysis of the gut microbiota, *bacteroidota* and *firmicutes* were two important intestinal flora. Therefore, we further analyzed the relative abundance of *firmicutes* and *bacteroidota* among Control, Model, and YQWYF groups in different levels (Figs. 8 and 9). The most *bacteroides* abundance of DKD mice in Model group were increased at the phylum (Fig. 8A), class (Fig. 8B), order (Fig. 8C), family (Fig. 8D), and genus (Fig. 8E) levels; namely, p-*bacteroidota*, c_*bacteroidia*, o_*bacteroidales*, f-*bacteroidaceae*, f_*prevotellaceae*, f-*tannerellaceae*, g_*alloprevotella*, g_*prarbacteroides*, and g_*bacteroides* in Model group were higher than those in Control group (*P* < 0.05). YQWYF obviously reduced the abundances of f_*prevotellaceae*, f-*tannerellaceae*, and g_*alloprevotella* compared with those in Model group (*P* < 0.05), and the abundances of p-*bacteroidota*, c_*bacteroidia*, o_*bacteroidales*, f_*muribaculaceae*, g_*prarbacteroides*, and g_*muribaculum* in YQWYF group were also lower than those in Model group; however, the differences were not statistically significant (*P* > 0.05). In particular, YQWYF continuously increased the relative abundance of g_*bacteroides* compared with that in Model group. The above results showed that YQWYF improved the relative abundances of abnormal *bacteroides* microbiota in different levels.

The most *firmicutes* abundance of DKD mice in Model group were decreased at the phylum (Fig. 9A), class (Fig. 9B), order (Fig. 9C), family (Fig. 9D), and genus (Fig. 9E) levels. In particular, p_*firmicutes*, c_*clostridia*, c_*firmicutes*, o_*lachnospirales*, o_*oscillospirales*, f_*ruminococcaceae*, f_*lachnospiraceae*, f_*[clostridium]_methylpentosum_group*, f_*peptococcaceae*, g_*colidextribacter*, g_*UCG-003*, g_*christensenellaceae_R-7_group*, g_*unidentified oscillospiraceae*, g_*[eubacterium]_ventriosum_group*, g_*[eubacterium]_xylanophilum_group*, g_*UCG-005*, and g_*oscillibacter* in Model group were lower than those in Control group (*P* < 0.05), and g_*facklamia* was higher than that in Control group (*P* < 0.05). YQWYF significantly increased the relative abundances of c_*clostridia*, o_*lachnospirales*, o_*peptococcales*, f_*ruminococcaceae*, f_*lachnospiraceae*, f_*[clostridium]_methylpentosum_group*, f_*peptococcaceae*, g_*colidextribacter*, g_*UCG-003*, g_*christensenellaceae_R-7_group*, and g_*[eubacterium]_ventriosum_group* compared with those in Model group (*P* < 0.05). Other bacteria also increased except g_*facklamia*, however, they had no significant differences between Model and YQWYF groups. These results showed that YQWYF improved the abundance of abnormal *firmicutes* microbiota in different levels.

### YQWYF improved jejunum and ileum histopathology in DKD mice

**Fig. 10:**
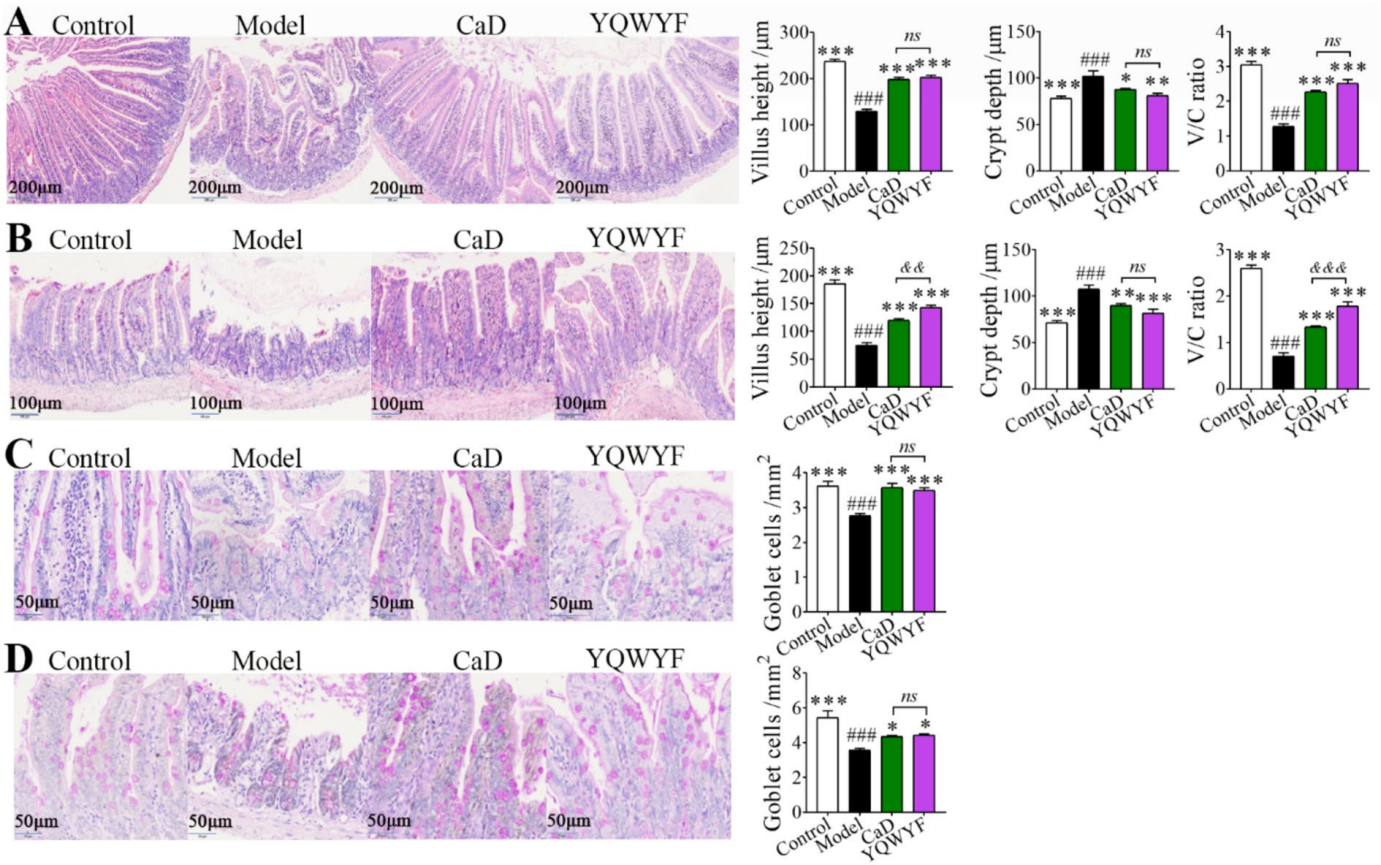
YQWYF improved jejunum and ileum histopathology in DKD mice (40 ×). **A** HE staining of the jejunum and statistical analysis of the villus height, crypt depth and villus height/crypt depth (V/C) ratio (*n* = 8). **B** HE staining of ileum and statistical analysis of the villus height, crypt depth and V/C ratio (*n* = 8). **C** PAS staining of jejunum and number of goblet cells (*n* = 8). **D** PAS staining of ileum and number of goblet cells (*n* = 8). The data are expressed as mean ± SEM, ^*^P < 0.05, ^**^P < 0.01, and ^***^P < 0.001 compared to Model group; ^###^*P* < 0.001 compared to Control group. ^&&^*P* < 0.01, and ^&&&^*P* < 0.001 compared to CaD group; *ns* showed no significance between two groups

STZ caused damage to the jejunum (Fig. 10A and C) and ileum (Fig. 10B and D), including an increase in crypt depth and a decrease in villus height, V/C ratio, and goblet cell number compared with Control group (Fig. 10*P* < 0.001). Compared with Model group, YQWYF and CaD significantly increased the villus height and V/C ratio and decreased the crypt depth of the jejunum and ileum (Fig. 10A and B, *P*  < 0.05), whereas YQWYF and CaD obviously increased the number of goblet cells in the jejunum and ileum (Fig. 10C and D, *P* < 0.05). The villus height and V/C ratio of ileum in YQWYF group were higher than those in CaD group (Fig. 10B, *P* < 0.01). These results showed that YQWYF improved histological injury of the jejunum and ileum.

### YQWYF alters the levels of bile acids in DKD mouse feces

**Fig. 11:**
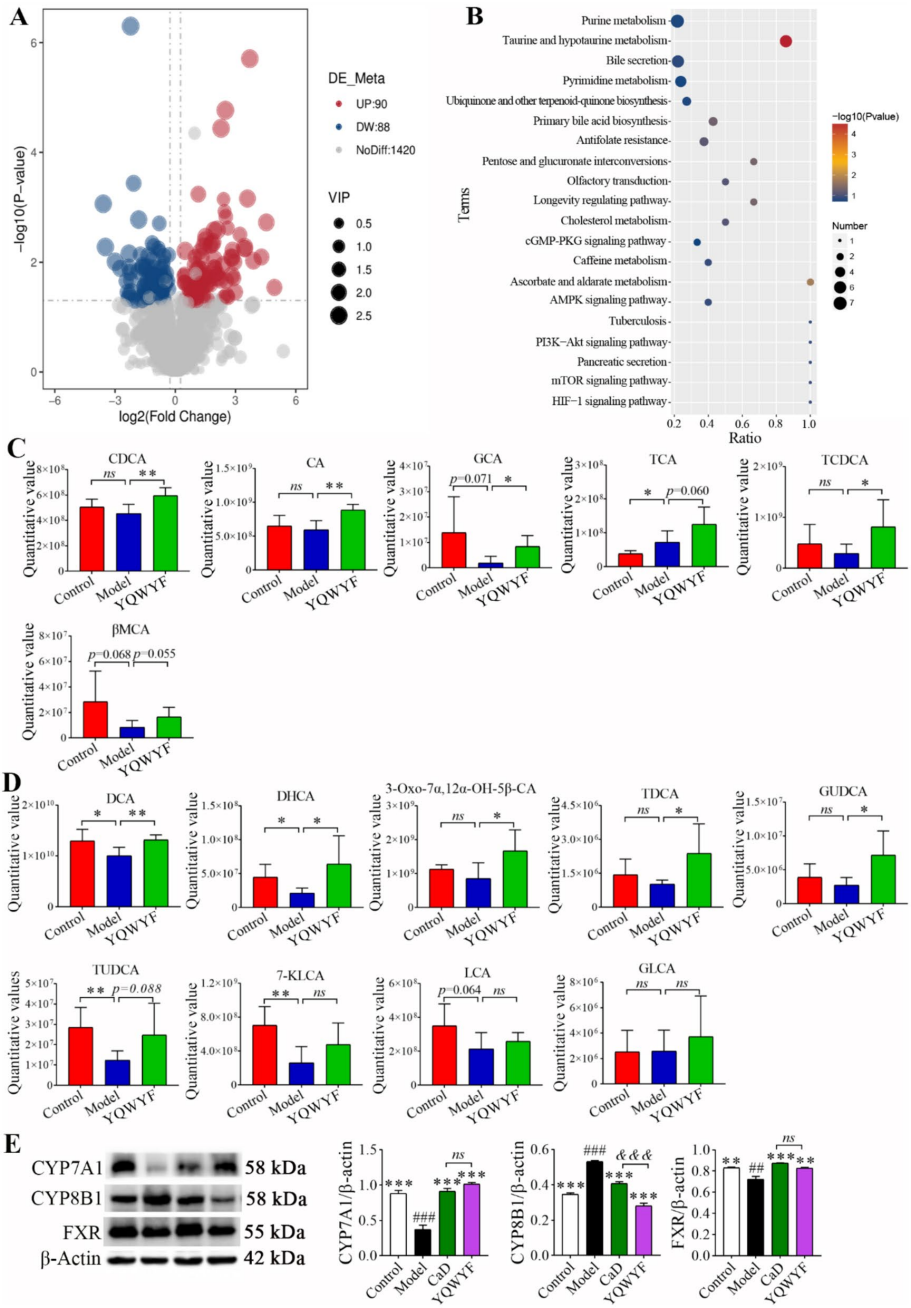
YQWYF alters bile acid levels in DKD mouse feces. **A** The volcano plot of differentially expressed metabolites between YQWYF and Model groups (*n* = 8). Red and blue circles indicate the significantly up- and down-regulated metabolites, respectively (|Fold change|> 2, *P* < 0.05). **B** The KEGG Enrichment analysis of 178 differential metabolites between YQWYF and Model group (*n* = 8). **C** Primary bile acid (*n* = 8). **D** Second bile acid (*n* = 8). **E** BAs metabolism-related enzymes expression in the liver (*n* = 6). The data are expressed as mean ± SEM, ^*^P < 0.05, ^**^P < 0.01, and ^***^P < 0.001 compared to Model group; ^##^*P* < 0.01 and ^###^*P* < 0.001 compared to Control group; ^&&&^*P* < 0.001 compared to CaD group; *ns* showed no significance between two groups

178 differential metabolites (including 90 up-regulated and 88 down-regulated) were identified between YQWYF and Model groups using untargeted metabolite (Fig. 11A). The results of top 20 KEGG Enrichment analysis showed that differential metabolites mainly enriched in bile acid metabolism related pathways including Taurine and hypotaurine metabolism, Bile secretion, and Primary bile acid biosynthesis (Fig. 11B). Bile acids (BAs) are important ingredients in the intestines and feces of hosts that maintain healthy gut contents and endocrine functions and control lipid absorption and the immune response [23]. The levels of most primary bile acid (Fig. 11C) and second bile acid (Fig. 11D) in Model group were decreased compared to Control group expect TCA. Especially, DCA, DHCA, TUDCA, and 7-KLCA were obviously reduced in Model group (Fig. 11D, *P* < 0.05). All bile acids of model mice treated with YQWYF for 18 weeks were increased compared to Model group. YQWYF significantly increased the levels of primary bile acids including CDCA, CA, GCA, and TCDCA (Fig. 11C, *P* < 0.05), and second bile acids including DCA, DHCA, 3-Oxo-7α,12α-OH-5β-CA, TDCA, and GUDCA (Fig. 11D, *P* < 0.05). Other bile acids also increased in YQWYF group, including TCA, βMCA, TUDCA, 7-KLCA, LCA, and GLCA; however, there were no significant differences between Model and YQWYF groups (*P* > 0.05). These results showed that YQWYF could regulate bile acid imbalance in DKD mouse feces.

CA is synthesized from cholesterol in the liver, and stands as one of the most prevalent components of BAs [24]. CYP7A1 and CYP8B are the key enzymes for regulating the synthesis of BAs; and Farnesoid X receptor (FXR) serves as a master regulator of BA metabolism [25]. Compared with Control group, the expressions of CYP7A1 and FXR was decreased, and the expression of CYP8B1 was increased in Model group (Fig. 11E, *P*< 0.01). In drug administration, YQWYF and CaD obviously increased the expressions of CYP7A1 and FXR, and decreased the expression of CYP8B1. Moreover, the expression of CYP8B1 in YQWYF group was lower than that in CaD group (Fig. 11E, *P* < 0.001). These results showed that YQWYF regulated BAs homeostasis and improving the expression of CYP7A1 and CYP8B1 in DKD mice by activating FXR signaling pathway.

### Correlation analysis of inflammatory factors, biochemical indicators, gut microbiota, and BAs

**Fig. 12:**
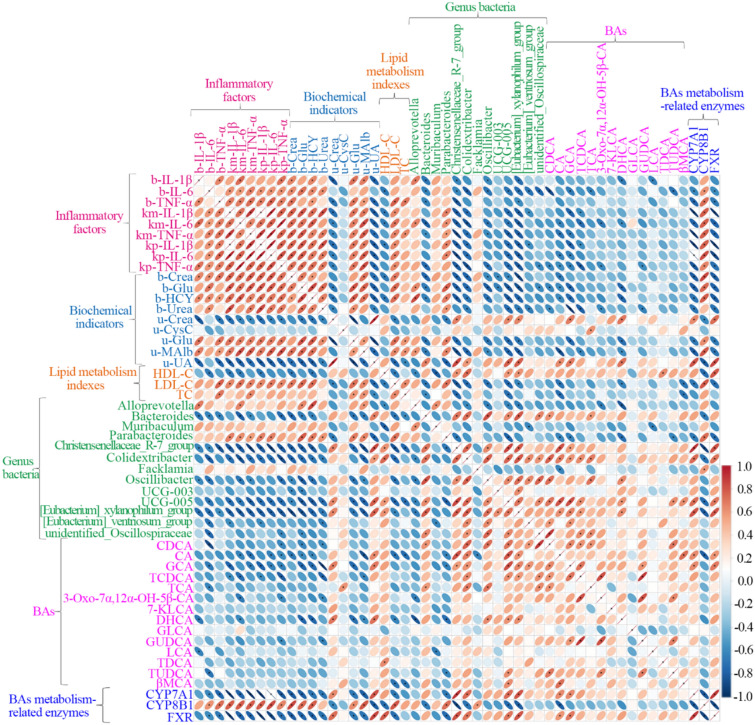
Spearman’s correlation analysis between Model and YQWYF groups. Correlation statistics were analyzed using the Spearman method. Red, positive association between nodes; blue, negative association between nodes. The data are expressed as mean ± SEM (*n* = 6), ^*^P < 0.05 compared to Model group

Data after normalization by log2 were used to analyze the correlations between each other (3 inflammatory factors, 9 biochemical indicators, 3 lipid metabolism indexes, 13 genus bacteria, 14 BAs, and 3 BAs metabolism-related enzymes) by Spearman’s correlation analysis. As shown in Fig. 12, b-IL-6, and b-TNF-α were positively correlated significantly with km-IL-1β, km-IL-6, km-TNF-α, kp-IL-1β, kp-IL-6, and kp-TNF-α. Namely, the increase of IL-6 and TNF-α in the blood promoted the mRNA and protein levels of IL-1β, IL-6 and TNF-α in the kidney. Levels of inflammatory factors in the blood and kidney were positively correlated with b-Crea, b-Glu, b-HCY, b-Urea, u-Glu, u-Malb and LDL-C, and were negatively correlated with u-Crea, u-UA and HDL-C. Therefore, IL-1β, IL-6 and TNF-α caused the changes of biochemical indicators in the blood and urine, and lipid metabolism. b-IL-1β, b-IL-6, and b-TNF-α were negatively correlated with *bacteroides*, *christensenellaceae_R-7_group*, and *[Eubacerium]_xylanophilum_group* in gut microbiota. Meanwhile, b-IL-6, and b-TNF-α significantly influenced the protein expressions of CYP7A1, CYP8B1, and FXR in the liver. The above results revealed that inflammatory factors were involved mainly in abnormal metabolism of glycometabolism, lipid metabolism, and gut microbiota.

In the biochemical indicators of serum, b-Glu and b-HCY were positively correlated with *alloprevotella* and *parabacteroides*; b-Crea, b-Glu and b-HCY were negatively correlated with *bacteroides*, *christensenellaceae_R-7_group*, *colidextribacter*, *oscillibacter*, *UCG-005*, and *[eubacerium]_xylanophilum_group* between the biochemical indicators of serum and gut microbiota (Fig. 12). In the biochemical indicators of urine, u-Crea and u-UA were positively correlated with *christensenellaceae_R-7_group*, *UCG-005*, and *[eubacerium]_xylanophilum_ group*, and negatively correlated with *parabacteroides*. u-Glu and u-MAlb were negatively correlated with *bacteroides*, *christensenellaceae_R-7_group*, *colidextribacter*, *oscillibacter*, *UCG-005*, and *[eubacerium]_xylanophilum_group*, and positively correlated with *parabacteroides* between the biochemical indicators of urine and gut microbiota (Fig. 12). In the lipid metabolism indexes of serum, HDL-C was positively correlated with *christensenellaceae_R-7_group*, *colidextribacter*, *UCG-005* and *[eubacerium]_xylanophilum_group*, and negatively correlated with *alloprevotella* and *muribaculum*. LDL-C was negatively correlated with *bacteroides*, *christensenellaceae_R-7_group*, *colidextribacter*, *oscillibacter*, *UCG-005* and *[eubacerium]_xylanophilum_group*, and positively correlated with *parabacteroides*. TC was negatively correlated with *bacteroides*, *christensenellaceae_R-7_group*, and *UCG-005* (Fig. 12). The above results revealed that the relative abundance of the gut microbiota was closely related to blood glucose, urine glucose and lipid metabolism in DKD mice.

There was close correlation between 10 BAs and 12 bacteria genera (Fig. 12). For example, the abundance of *parabacteroides* was negatively correlated with CA, GCA, DHCA, and TUDCA; CA and GCA were positively correlated with *christensenellaceae_R-7_group*, *colidextribacter*, *UCG-005* and *[eubacerium]_xylanophilum_group*; CDCA and TCA were positively correlated with *bacteroides*, *colidextribacter*, *oscillibacter* and *unidentified_oscillospiraceae*. Importantly, 3 genus bacteria (*parabacteroides*, *UCG-005* and *[eubacerium]_xylanophilum_group*) were closely related with 3 BAs (CA, GCA and DHCA) in DKD mice. Therefore, we thought that the gut microbiota regulated BA metabolism in DKD mice.

For the biochemical indicators and BA analysis (Fig. 12), b-Glu was negatively correlated with 4 BAs (CDCA, CA, GCA, and DHCA), b-HCY was negatively correlated with 3 BAs (CA, GCA, and DHCA), and b-Urea was negatively correlated with 5 BAs (CDCA, GCA, TCDCA, 3-Oxo-7α, 12α-OH-5β-CA, and GUDCA). In the analysis of urine biochemical indicators and BAs, u-Glu was negatively correlated with GCA and DHCA, whereas u-MAlb was negatively correlated with CA, TCDCA, and DHCA. u-UA was positively correlated with CA, GCA and DHCA. Lipid metabolism indicator and BA analyses revealed that HDL-C was only positively correlated with TDCA; LDL-C was negatively correlated with CA, DHCA, and βMCA; and TC was negatively correlated with CA and βMCA. These results showed that BAs were involved in regulating the blood glucose, urine glucose and lipid metabolism of DKD mice.

Importantly, CYP7A1 and FXR were positively correlated with CA and GCA; and CYP8B1 was negatively correlated with CA. In gut microbiota, CYP7A1 and FXR were positively with *christensenellaceae_R-7_group*, *colidextribacter*, *UCG-005* and *[eubacerium]_xylanophilum_group*, and negatively correlated with *alloprevotella*. CYP8B1 was positively correlated with *parabacteroides* and *facklamia*, and negatively correlated with *colidextribacter*, *christensenellaceae_R-7_group*, *UCG-005* and *[eubacerium]_xylanophilum_group*. Meanwhile, CYP7A1 and FXR were negatively correlated with b-IL-6 and b-TNF-α, km-IL-1β, km-IL-6, km-TNF-α, kp-IL-1β, kp-IL-6, kp-TNF-α, b-Crea, b-Glu, b-HCY, u-Crea, u-Glu, u-MAlb, u-UA, and LDL-C; however, CYP8B1 was positively correlated with above indexes. Therefore, the protein expressions of CYP7A1, CYP8B1, and FXR in the liver had a close relationship with BAs, gut microbiota, inflammatory factors, glycometabolism and lipid metabolism.

## Discussion

**Fig. 13:**
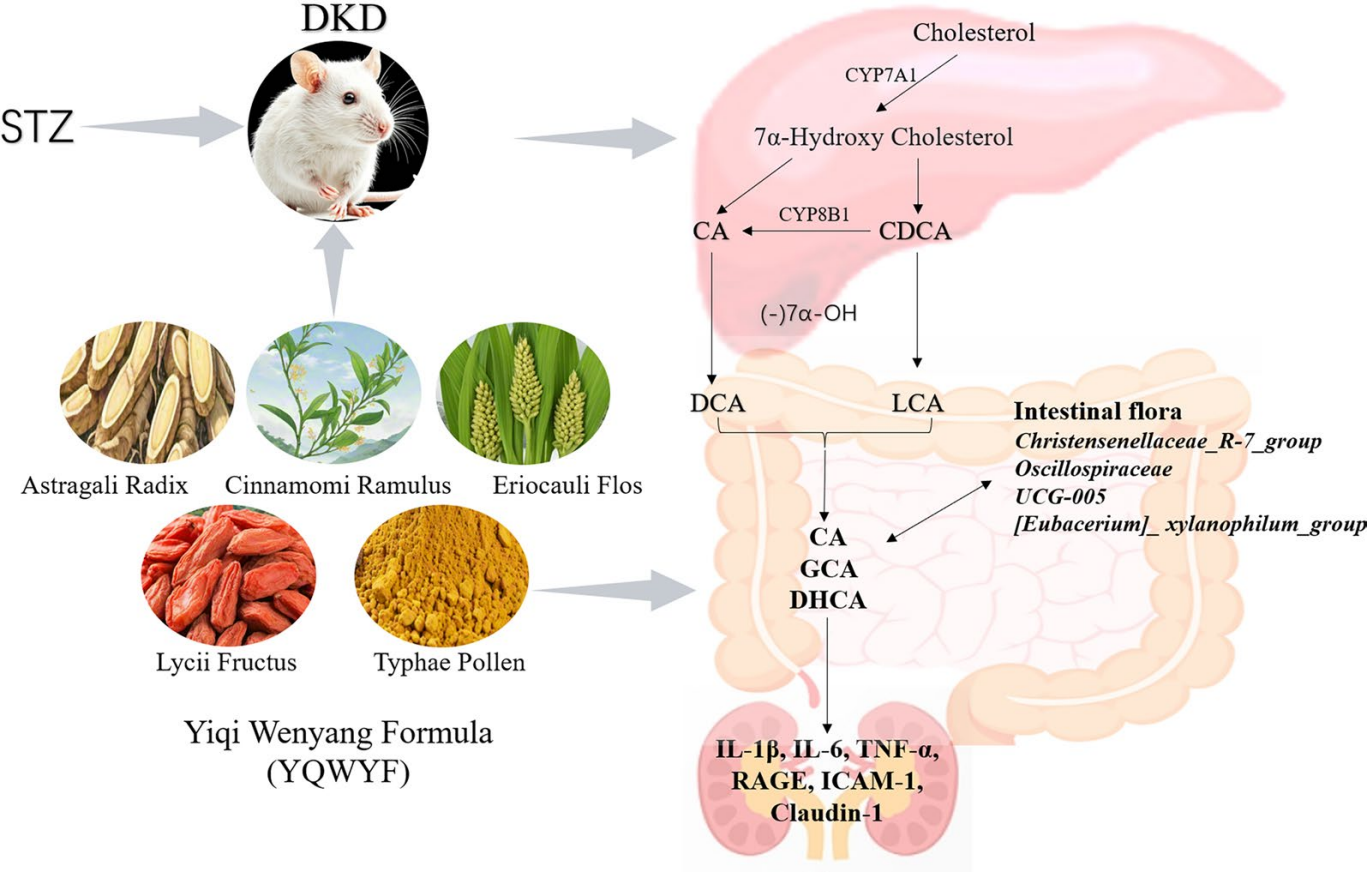
Graphical abstract

DKD is a microvascular complication of DM, and a long-term hyperglycemic environment causes hypertension, the later brings about the disturbance of renal perfusion pressure and further damages the microvasculature of renal arteries, glomerular and tubulointerstitial capillaries. In this study, a combination of network pharmacology and animal experiments was used to study the preventive and therapeutic effects of YQWYF on DKD mice. YQWYF included five Chinese medicinal herbs, that was Astragali Radix (AR), Lycii Fructus (LF), Eriocauli Flos (EF), Cinnamomi Ramulus (CR), and Typhae pollen (TP). Network pharmacology results revealed that TNF-α and IL-6, as important core targets, docked with 33 compounds in YQWYF. The experimental results in mice showed that YQWYF reduced the levels of b-Glu and u-Glu, inhibited inflammatory cytokines, kidney tissue factors and oxidative stress, recovered the richness and evenness of the gut microbiota, increased the bile acid levels in mouse feces (Fig. 13). These results suggest that YQWYF protects kidney and delays DKD development, further supporting the potential application of YQWYF in the treatment of DKD.

The results of UPLC-Q-TOF/MS detection showed that YQWYF contained 15 kinds of flavones and 10 kinds of glycosides among 41 compounds, which exerted anti-oxidant, anti-inflammatory effects [26], and improved glycose and lipid metabolic abnormalities [27, 28]. The top 10 core targets of YQWYF for DKD treatment were obtained by network pharmacology including TNF, IL6, AKT1, GAPDH, BCL2, ESR1, SRC, CTNNB1, EGFR, and PPARG. In GO and KEGG enrichment analysis, ‘PI3K-Akt signaling pathway’, ‘MAPK signaling pathway’, and ‘AGE-RAGE signaling pathway in diabetic complications’ were closely related to the pathological process of DKD. Astragaloside I, II and IV from Astragali Radix has been found to prevent and delay the occurrence and development of DKD [29]. Astragaloside I improved renal dysfunction and fibrosis in DKD mice by regulating the HDAC3/Klotho/TGF-β1 pathway [30]. Astragaloside II reduces albuminuria, improves renal histopathology and mitochondrial dysfunction, and inhibits podocyte apoptosis in DM rats by regulating the Nrf2 and PINK1 pathways [31]. Astragaloside IV inhibits oxidative stress, endoplasmic reticulum stress and inflammation; regulates calcium homeostasis; and improves vascular function to exhibited excellence protective effects on DKD, which is associated with multiple signaling pathways, such as the AMPK signaling pathway, NF-κB signaling pathway, and PI3K/Akt/mTOR signaling pathway [32, 33]. Calycosin and calycosin-7-O-β-D-glucoside from Astragali Radix also have protective effects on DKD mice by regulating ferroptosis [34]. Lycibarbarspermidines F, J, and M, lycibarbarphenylpropanoid A and glu-lycibarbarspermidine F have anti-oxidant, anti-aging and neuroprotective effects [35, 36]. The naringenin kaempferol, isorhamnetin, kaempferol-3-O-neohesperidoside and isorhamnetin-3-O-neohespeidoside from Typhae pollen are flavonoids that display strong anti-inflammatory, anti-oxidant and anti-fibrotic activities [37]. He et al. reported that naringenin ameliorated cardiac microvascular dysfunction in DM mice by regulating the Nrf2 and NF-κB signaling pathways [38]. The molecular docking results revealed that astragaloside I, astragalside IV, and 16-meprednisone acetate had the strong affinity with TNF-α and IL-6; isoastragaloside I had the strongest affinity with TNF-α; astragaloside II had the strongest affinity with IL-6; meanwhile, formononetin, calycosin, and naringenin also showed the strong affinity with IL-6. Using network pharmacology and to investigate the interactions among drug components, targets, and diseases revealed the potential mechanisms of YQWYF treatment for DKD. Furthermore, animal experiments validated the potential therapeutic effects of YQWYF on DKD.

DKD mice with random blood glucose concentrations ≥ 11.1 mmol/L induced by STZ. u-CysC, u-MAlb and b-Crea are biomarkers of DKD [39]. b-Crea and u-MAlb continue to increase in DKD patients [40]. Mogos et al. reported that UA from urine could reflect DKD and DM progression [41]. In our study, the levels of u-Crea and u-UA significantly decreased, whereas the levels of u-MAlb and u-CysC increased after 9 weeks in model mice induced by STZ. These indicators were abnormal until the end of the experiment (18 weeks). The above results showed that DKD mice were successfully induced by 50 mg/kg STZ for 5 days. CaD, as a positive drug, is effective drug for the treatment of diabetic microvascular complications [13]. CaD improved the endothelial dysfunction and inflammation caused by high glucose[42], and restored autophagy by inhibiting the VEGF/PI3K/AKT/mTOR signaling pathway[43]. Ozdemir et al. reported that CaD had anti-inflammatory, anti-oxidant, anti-apoptotic, and anti-autophagic effects [44], and improved renal injury in DKD mice [14]. Meanwhile, we found that CaD obviously reduced blood glucose [43]; however, urine glucose had decreased slightly compared to Model group. YQWYF significantly ameliorated the biochemical indicators in urine and serum; specifically, it could reduce the levels of u-Glu and b-Glu [45]. Our results suggested that YQWYF had hypoglycemic and renoprotective effects.

Many scholars increasingly conform that systemic inflammation is the common mechanism of macrovascular and microvascularin diseases in DM [3, 46]. High glucose causes vascular endothelial cell (EC) damage that give rise to inflammation mediators (inflammatory cytokines, adhesion molecules, chemokines, and growth factors) release, oxidative stress and advanced glycation end-products (AGEs) accumulation in cells and tissue, they promote kidney damage in DKD patients [3]. IL-1β activates NF-κB pathway that causes the apoptosis of capillary ECs and increases EC permeability [47]. Pang reported that Yiqi Tongluo Fang inhibited the release of IL-1β and TNF-α in the serum and reduced the expression of TNF-α, NF-κB p65, and p38MAPK in the retinas of DR mice [11]. Importantly, spearman correlation analysis showed that the increase of proinflammatory factor in the blood promoted the expressions of IL-1β, IL-6 and TNF-α in the kidney, caused the abnormal of lipid metabolism indexes and biochemical indicators in blood and urine, reduced the relative abundance of *christensenellaceae_R-7_group*,* oscillibacter* and *[eubacerium]_xylanophilum_group*, inhibited the expressions of CYP7A and FXR, and increased the expression of CYP8B1 in liver. Therefore, inflammation was an important factor that participated in regulation of glycometabolism, lipid metabolism, BAs metabolism and gut microbiota disorders [48]. However, YQWYF exhibits excellent anti-inflammatory effects in DKD mice.

AGEs are considered as key participants in the progression of diabetes and its complications. Uncontrolled hyperglycemia causes glucose and lipid metabolism disorder that generate lots of AGEs accumulation. AGEs may promote the production of ROS, NF-κB, IL-1β, IL-6, and TNF-α by interacting with receptors of AGEs (RAGE) on the cell surface, which induce the generation of abundant intracellular ROS, and initiate oxidative stress and inflammation cascades [3]. AGEs bind to some proteins, including laminin, elastin, and collagen, which increases vascular hardness and permeability. RAGE also promotes the release of growth factors and adhesion molecules (ICAM-1). The ectopic expression of claudin-1 is increased in damaged podocytes in DM and DKD [49, 50]. Our results showed that YQWYF reduced the expression of RAGE, ICAM-1 and Claudin-1 in the kidney, meanwhile, YQWYF also increased the levels of oxidoreductases (GSH, GPx, CAT, and SOD) and HDL-C, and decreased the oxidative tissue damage parameters (MDA and ROS), LDL-C and TC. Therefore, YQWYF may improve disorders of glucometabolic and lipid metabolism by inhibiting inflammation cascades and oxidative stress.

Gut microbiota dysbiosis has been shown to occur in DKD patients and mice [51]. The sequencing coverage rate, richness, and evenness of DKD children and mice are reduced [52, 53]. Our results showed that YQWYF recovered the abnormal abundance of the gut microbiota at the phylum, class, order, family, and genus levels. Spearman correlation analysis showed that *christensenellaceae_R-7_group*, *oscillibacter*, *UCG-005* and *[eubacerium]_xylanophilum_group* were negatively correlated with b-Crea, b-Glu, b-HCY, u-Glu, u-MAlb, and LDL-C; these genera were related to beneficial effects of YQWYF on DKD mice. Jin et al. reported that *christensenellaceae_R-7_group* was involved in the regulation of tryptophan metabolism to treat T2DM [54]. The increase of *oscillibacter* and *[eubacerium]_xylanophilum_group* enhanced the production of short-chain fatty acids to alleviating T2DM symptoms [55, 56]. Our results also found that *alloprevotella* and *parabacteroides* were positively correlated with b-Glu, b-HCY, and u-MAlb. Salman et al. reported that the increase of *alloprevotella* gave rise to childhood obesity and metabolic syndrome [57]. *Alloprevotella* and *parabacteroides* were harmful bacteria which participated in regulating glucose and lipid metabolism disorders [48]. Therefore, YQWYF ameliorated the blood and urine glucose, and renal injury in DKD mice by regulating the gut microbiota.

BAs play important roles in maintaining host nutrient metabolism and energy, which participate in regulating immune function, digestion and absorption of lipids and glycometabolism [58]. Some researchers reported that the loss or imbalance of primary BAs and second BAs in the gut and blood was associated with inflammation and diabetes development and progression [23, 59]. Cytochrome P450 enzymes CYP7A1 and CYP8B1 play crucial roles in bile acid synthesis, which are fundamental for maintaining normal liver function, cholesterol metabolism, and overall digestive health [60]. The nuclear farnesoid X receptor (FXR), the major BA receptors, can maintain within a physiological range of BAs to support normal digestive function and prevent liver and intestinal diseases associated with bile acid dysregulation [61]. FXR also participate in regulating glucose metabolism [62]. Our results showed that the levels of most primary BAs and second BAs of feces in DKD mice were decreased compared to Control group except TCA, which is consistent with the reported [63]. Gut microbiota regulated bile acid metabolism via gut-liver axis [64]. Spearman correlation analysis revealed that CYP7A1, CYP8B1 and FXR were closely correlated with BAs (CA and GCA) and bacteria genera (*parabacteroides*, *christensenellaceae_R-7_group*, *colidextribacter*, *UCG-005* and *[eubacerium]_xylanophilum_group*). Meanwhile, CA and GCA were positively correlated with *christensenellaceae_R-7_group*, *colidextribacter*, *UCG-005* and *[eubacerium]_xylanophilum_group*. However, 4 BAs (GCA, 3-Oxo-7α,12α-OH-5β-CA, DHCA, and TUDCA) were negatively correlated with *parabacteroides*. CA, GCA and DHCA were important BAs in DKD mice, they participated in improving glycometabolism, and lipids metabolism [65]. YQWYF significantly increased the levels of CA, CDCA, TCDCA, GCA, DCA, DHCA, 3-Oxo-7α,12α-OH-5β-CA, TDCA and GUDCA in DKD mouse feces. Meanwhile, YQWYF increased the expression of CYP7A1 and FXR, and decreased the expression of CYP8B1. These results indicated that YQWYF improved renal injury via regulating the gut microbiota-BA axis in DKD mice by FXR signaling pathway.

## Conclusion

41 compounds were identified from YQWYF, and three components of YQWYF (astragaloside I, 16-meprednisone acetate, and astragaloside IV) may be pivotal in reducing the inflammatory cascade by network pharmacology and molecular docking. YQWYF improved glycometabolism, lipid metabolism, intestinal homeostasis, and pathological changes in DKD mice by inhibiting the inflammatory response and regulating the gut microbiota-BA axis. Specifically, YQWYF restrained the levels of IL-1β, IL-6, TNF-α, RAGE, ICAM-1 and Claudin-1 in the kidney, and recovered the composition of abnormal microbiota and BA levels. Importantly, 4 genus bacteria (*christensenellaceae_R-7_group*, *oscillibacter*, *UCG-005* and *[Eubacerium]_xylanophilum_group*) were closely related with 3 BAs (CA, GCA and DHCA), and they play a very important role in ameliorating DKD via regulating the gut microbiota-BA axis in mice.

## Supplementary Information

Additional file 1 (DOCX 34 KB)

## Data Availability

The data used in this study can be provided in accordance with requests.

## Abbreviations

3-Oxo-7α, 12α-OH-5β-CA: 3-Oxo-7alpha, 12alpha-hydroxy-5beta-cholanoic acid
7-KLCA: 7-Ketolithocholic acid
βMCA: β-Muricholic acid
AR: Astragali Radix
ASVs: Amplicon sequence variants
BA: Bile acid
b-Crea: Creatinine in the blood
b-Glu: Glucose in the blood
b-Hcy: Homocysteine in the blood
b-Urea: Urea in the blood
CA: Cholic acid
CaD: Calcium dobesilate
CAT: Catalase
CDCA: Chenodeoxycholic acid
CR: Cinnamomi Ramulus
DAVID: Database for annotation, visualization, and integrated discovery
DCA: Deoxycholic acid
DHCA: Dehydrocholic acid
DKD: Diabetic kidney disease
DM: Diabetes mellitus
EF: Eriocauli Flos
ESI: Electrospray ionization
GCA: Glycocholic acid
GLCA: Glycolithocholic acid
GO: Gene ontology
GPx: Glutathione peroxidase
GSH: Glutathione
GUDCA: Glycoursodeoxycholic acid
HDL-C: High-density lipoprotein cholesterol
HE: Hematoxylin-eosin
IL-1β: Interleukin-1 beta
IL-6: Interleukin-6
IM-QTOF: Ion mobility-quadrupole-time of flight
IP: Intraperitoneal injection
KEGG: Kyotoencyclopedia of genes and genomes
km-IL-1β: MRNA level of interleukin-1 beta in the kidney
km-IL-6: MRNA level of interleukin-6 in the kidney
km-TNF-α: MRNA level of tumor necrosis factor-α in the kidney
kp-IL-1β: Protein expression of interleukin-1 beta in the kidney
kp-IL-6: Protein expression of interleukin-6 in the kidney
kp-TNF-α: Protein expression of tumor necrosis factor-α in the kidney
LCA: Lithocholic acid
LC-MS: Liquid chromatograph-mass spectrometer
LDH: Lactate dehydrogenase
LDL-C: Low-density lipoprotein cholesterol
LF: Lycii Fructus
MDA: Malondialdehyde
PAS: Periodic-acid Schiff
PPI: Protein-protein interaction
PVDF: Polyvinylidene fluoride
ROS: Reactive oxygen species
RT qPCR: Real-time quantitative PCR
SDS-PAGE: Sodium dodecyl sulfate–polyacrylamide gel electrophoresis
SEM: Standard error of the mean
SOD: Superoxide dismutase
STZ: Streptozocin
T1D: Type 1 diabetes
T2D: Type 2 diabetes
TC: Total cholesterol
TCA: Taurocholic acid
TCDCA: Taurochenodeoxycholic acid
TCMSP: Traditional Chinese medicine systems pharmacology database
TDCA: Taurodeoxycholic acid
TNF-α: Tumor necrosis factor-α
TP: Typhae Pollen
TUDCA: Tauroursodeoxycholic acid
u-Crea: Creatinine in the urine
u-CysC: Cystatin C in the urine
u-Glu: Glucose in the urine
u-MAlb: Microalbumin in the urine
u-UA: Uric acid in the urine
UPLC: Ultra-high performance liquid chromatography
UPLC-Q-TOF/MS: Ultra-high performance liquid chromatography/ quadrupole time-of-flight mass spectrometry
YQWYF: Yiqi Wenyang Formula
