# Supplementary material for: Yiqi Wenyang Formula ameliorates diabetic kidney disease via inhibiting inflammation and regulating the gut microbiota-bile acid axis in mice by FXR signaling pathway
Source: Chin Med. 2025 Oct 30;20:183. doi: 10.1186/s13020-025-01238-3 (PMC12574177; doi:10.1186/s13020-025-01238-3)
Supplement: Supplementary file 1 — Additional file 1 (DOCX 34 KB) [file 13020_2025_1238_MOESM1_ESM.docx]

Supplementary Table S1. The compounds were identified from YQWYF by UHPLC/QTOF-MS

| **No.** | **RT (min)** | **Formula** | **Negative ion mode(-)** | | | |  | **Positive ion mode(+)** | | | | **Identification** | **Source** |
| --- | --- | --- | --- | --- | --- | --- | --- | --- | --- | --- | --- | --- | --- |
|  |  |  | **Observed *m/z*** | **Adducts** | **Mass error (ppm)** | **MS/MS(-)** |  | **Observed *m/z*** | **Adducts** | **Mass error (ppm)** | **MS/MS(+)** |  |  |
| 1 | 3.49 | C_10_H_13_O_4_N_5_ | / | | | |  | 268.1041 | [M+H]^+^ | 0.37 | 268.1035,136.0616,119.0348 | Adenosine | AR |
| 2 | 3.99 | C_10_H_13_O_5_N_5_ | 282.084 | [M-H]^-^ | -1.77 | 282.0837,267.0721,205.9766,150.0451,135.0307,133.0151 |  | 284.0978 | [M+H]^+^ | -3.89 | 284.0990,152.0564,135.0296 | Guanosine | AR |
| 3 | 5.42 | C_9_H_11_NO_2_ | / | | | |  | 166.0857 | [M+H]^+^ | -3.03 | 166.0860,149.0595,120.0805,103.0540 | L-Phenylalanine | AR |
| 4 | 6.7 | C_18_H_26_O_12_ | 433.1334 | [M-H]^-^ | -4.15 | 433.1354,401.1322,385.1251,355.1628,344.1818,279.1340,259.1290,241.1195,124.0161 |  | / | | | | Markhamioside F | AR |
| 5 | 6.83 | C_18_H_24_O_12_ | 431.1195 | [M-H]^-^ | -0.23 | 431.1199,323.0986,221.0663,161.0452,137.0241,133.0153 |  | / | | | | Asperulosidic acid | AR |
| 6 | 7.41 | C_21_H_28_O_13_ | 487.1457 | [M-H]^-^ | -0.20 | 267.9827,237.9934,221.9856,205.9911,119.0497 |  | / | | | | Lycibarbarphenylpropanoid A | LF |
| 7 | 7.50 | C_11_H_11_NO_3_ | / | | | |  | 188.0700 | [M-H_2_O+H]^+^ | -2.67 | 188.0723,158.0599,158.0599,146.0599,142.0648,118.0649,117.0571,115.0540 | Indolelactic acid | AR |
| 8 | 7.84 | C_37_H_55_O_16_N_3_ | / | | | |  | 798.3623 | [M+H]^+^ | -3.89 | 676.3136,474.2596,384.1649,222.1119,165.0541 | Lycibarbarspermidine M | LF |
| 9 | 8.08 | C_37_H_53_O_16_N_3_ | 794.3338 | [M-H]^-^ | -2.01 | 795.3384,417.1047,335.1802,313.0495,291.1708,239.0125,201.0663,135.0449 |  | 796.3460 | [M+H]^+^ | -4.78 | 634.2966,472.2431,310.2120,220.0966,163.0388 | Lycibarbarspermidine F | LF |
| 10 | 8.17 | C_31_H_45_O_11_N_3_ | / | | | |  | 636.3113 | [M+H]^+^ | -2.05 | 474.2587,384.1643,317.0651,222.1120,163.0387 | Lycibarbarspermidine J | LF |
| 11 | 8.25 | C_43_H_63_O_21_N_3_ | / | | | |  | 958.4003 | [M+H]^+^ | -2.40 | 634.2963,472.2443,382.1501,310.2123,303.0494,220.0965,163.0783 | Glu-lycibarbarspermidine F | LF |
| 12 | 9.65 | C_16_H_12_O_6_ | / | | | |  | 301.0693 | [M+H]^+^ | -4.33 | 301.0705,269.0438,241.0490,213.0550,211.0861,187.0384,128.0620 | Rhamnocitrin | AR |
| 13 | 9.91 | C_33_H_40_O_20_ | 755.2038 | [M-H]^-^ | -0.4 | 755.2042,413.9687,271.0243,255.0294,243.0295,151.0033 |  | / | | | | Quercetin-3-*O*-rutinoside-(1-2)-*O*-rhamnoside | TP |
| 14 | 10.15 | C_9_H_8_O_3_ | 163.0400 | [M-H]^-^ | -0.61 | 163.0400,120.0540,119.0496 |  | / | | | | *p*-Coumaric acid | TP |
| 15 | 10.81 | C_34_H_42_O_20_ | 769.2169 | [M-H]^-^ | -3.64 | 769.2196,605.1509,299.0191,271.0240,243.0290,227.0343,199.0394 |  | / | | | | Typhaneoside | TP |
| 16 | 10.88 | C_10_H_8_O_4_ | / | | | |  | 193.0488 | [M+H]^+^ | -3.65 | 187.9777,178.0256,149.0231,133.0281,121.0281,105.0332,102.9932 | Scopoletin | LF |
| 17 | 10.98 | C_22_H_22_O_10_ | 491.1192 | [M-H+FA]^-^ | -0.52 | 491.1182,445.1193,283.0607,268.0370,211.0394,195.0447 |  | 447.127 | [M+H]^+^ | -3.36 | 447.1287,285.0752,213.0544,197.0292,157.0645,137.0230,115.0540 | Calycosin-7-*O*-β-D-glucoside | AR |
| 28 | 11.44 | C_27_H_30_O_15_ | 593.1514 | [M-H]^-^ | 0.36 | 593.1520,285.0388,255.0295,227.0347 |  | 595.1647 | [M+H]^+^ | -1.68 | 595.1656,449.1078,340.9733,288.0582,287.0544,153.0181 | Kaempferol-3-*O*-neohesperidoside | TP |
| 29 | 11.61 | C_28_H_32_O_16_ | 623.1616 | [M-H]^-^ | -0.33 | 623.1610,503.1194,459.0934,299.0191,271.0240,243.0290,199.0395,171.0448 |  | 625.1745 | [M+H]^+^ | -2.72 | 625.1760,317.0650,285.0385,245.0438,229.0485,217.0492,201.0541,153.0179 | Isorhamnetin-3-*O*-neohespeidoside | TP |
| 20 | 12.46 | C_28_H_32_O_16_ | 623.1618 | [M-H]^-^ | 0.1 | 623.1619,315.0504,299.0194,271.0243,243.0293 |  | 625.1742 | [M+H]^+^ | -3.20 | 625.1757,317.0648,302.0411,285.0386,245.0434,229.0487,205.0751,153.0181 | Narcissoside | TP/LF |
| 21 | 14.20 | C_22_H_22_O_9_ | 475.1246 | [M-H+FA]^-^ | -0.06 | 267.0659,223.0396,195.0446,167.0494 |  | 431.1325 | [M+H]^+^ | -2.56 | 431.1350,269.0806,253.0492,225.0542,197.0593,181.0643,152.0615,138.0547,115.0538 | Ononin | AR |
| 22 | 14.75 | C_23_H_28_O_10_ | 463.1613 | [M-H]^-^ | 0.65 | 301.1071,283.1071,211.0390,167.0495 |  | / | | | | Astraisoflavan-7-*O*-β-D-glucoside | AR |
| 23 | 14.82 | C_15_H_12_O_5_ | / | | | |  | 273.0744 | [M+H]^+^ | -2.82 | 285.0753,213.0542,128.0616,115.0540 | Calycosin | AR |
| 24 | 15.01 | C_17_H_14_O_6_ | / | | | |  | 315.0863 | [M+H]^+^ | 0.32 | 315.0857,308.2214,299.0543,285.0753,283.0587,184.0513,171.0438,155.0490,115.0541 | Jaranol | AR |
| 25 | 15.58 | C_15_H_12_O_5_ | 271.0608 | [M-H]^-^ | -1.84 | 271.0608,177.0190,151.0034,147.0813,121.0656,119.9283 |  | 273.0744 | [M+H]^+^ | -4.78 | 273.0755,269.0808,253.0491,237.0544,207.1195,197.0598,181.0646,153.0181,131.0853,107.0488 | Naringenin | TP |
| 26 | 15.82 | C_15_H_10_O_6_ | / | | | |  | 287.0554 | [M+H]^+^ | 1.75 | 287.0551,257.0443,213.0544,155.0492,153.0180,128.0616,115.0540 | Kaempferol | TP |
| 27 | 16.05 | C_16_H_12_O_7_ | 315.0513 | [M-H]^-^ | 0.63 | 313.0354,285.2074,271.0241,227.0346,185.0238,163.0038 |  | 317.0657 | [M+H]^+^ | 0.63 | 317.0650,287.0546,245.0441,217.0497,207.1198,203.0335,153.0183 | Isorhamnetin | TP |
| 28 | 16.27 | C_47_H_78_O_19_ | 991.5128 | [M-H+FA]^-^ | 0.85 | 945.5069,813.4493,783.4543,765.4432,621.4011,489.3586,383.2955 |  | / | | | | Astragaloside VII | AR |
| 29 | 17.08 | C_41_H_68_O_14_ | 829.4588 | [M-H+FA]^-^ | -0.42 | 829.4570,783.4520,621.4000,489.3586,265.0860,237.0909,179.0553,159.0442 |  | 785.4674 | [M+H]^+^ | -0.89 | 587.3947,455.3524,437.3417,419.3312,297.2210,143.1064 | Astragaloside Ⅳ | AR |
| 30 | 17.24 | C_16_H_12_O_4_ | 267.0656 | [M-H]^-^ | -2.42 | 267.0654,223.0391,195.0444,167.0495 |  | 269.0802 | [M+H]^+^ | -2.24 | 269.0804,267.0651,253.0294,225.0544,197.0593,181.0647,139.0542,115.0540 | Formononetin | AR |
| 31 | 17.42 | C_41_H_68_O_14_ | 829.4586 | [M-H+FA]^-^ | -0.62 | 783.4528,621.4009,489.3583,383.2952,161.0450 |  | / | | | | Astragaloside III | AR |
| 32 | 19.13 | C_43_H_70_O_15_ | 871.4693 | [M-H+FA]^-^ | -0.41 | 871.4684,825.4637,783.4537,765.4433 |  | 827.4792 | [M+H]^+^ | 0.60 | 653.4226,455.3523,437.3409,419.3320,223.1475,207.1169,165.0698128.0615,115.0543,107.0851 | Astragaloside II | AR |
| 33 | 19.85 | C_43_H_70_O_15_ | 871.4701 | [M-H+FA]^-^ | 0.51 | 871.4689,825.4636,783.4550,765.4430 |  | / | | | | Isoastragaloside | AR |
| 34 | 19.89 | C_48_H_78_O_18_ | / | | | |  | 943.5225 | [M+H]^+^ | -3.71 | 617.4047,599.3932,441.3730,423.3624，405.3512,269.2261,203.1791 | Soyasaponin I | AR |
| 35 | 20.74 | C_45_H_72_O_16_ | 913.4813 | [M-H+FA]^-^ | 1.15 | 913.4812,867.4756,529.2479,357.2646,313.2378,311.2228,223.1700,195.1031,171.1025,117.9287 |  | 869.4862 | [M+H]^+^ | -3.45 | 869.4870,875.4950,653.4043,509.2716,455.3522,437.3413,419.3309,297.2210,217.0702,157.0492 | Astragaloside I | AR |
| 36 | 21.07 | C_45_H_72_O_16_ | 913.4804 | [M-H+FA]^-^ | 0.12 | 913.4801,867.4738,443.2803,417.2641,313.2381,311.2224,293.2120,265.1469,117.9283 |  | 869.4861 | [M+H]^+^ | -3.57 | 711.4088,695.4353,437.3415,419.3304,209.1320,195.1153,183.1168,169.0493,165.0699,125.0957,107.0852 | Isoastragaloside I | AR |
| 37 | 21.37 | C_24_H_30_O_6_ | / | | | |  | 415.2101 | [M+H]^+^ | -3.14 | 318.3001,282.2788,254.1409,120.0889,119.0853,117.0696,115.0540,103.0537 | 16-Meprednisone acetate | CR/LF |
| 38 | 22.14 | C_18_H_28_O_2_ | / | | | |  | 277.2156 | [M+H]^+^ | -1.81 | - | 12-Phenyldodecanoic acid | AR/TP |
| 39 | 22.78 | C_18_H_32_O_3_ | 295.2276 | [M-H]^-^ | -1.01 | 295.2276,277.2169 |  | / | | | | 9-Hydroxy-10,12-octadecadienoic acid | AR/TP |
| 40 | 22.8 | C_18_H_30_O_2_ | / | | | |  | 279.2312 | [M+H]^+^ | -2.16 | - | Linoleic acid | TP |
| 41 | 26.3 | C_19_H_36_O_3_ | / | | | |  | 313.2742 | [M+H]^+^ | 1.92 | 313.2734,307.1722,305.1571,303.1402 | Methyl ricinolate | AR/TP |
